# Methylation of Subtelomeric Chromatin Modifies the Expression of the lncRNA TERRA, Disturbing Telomere Homeostasis

**DOI:** 10.3390/ijms23063271

**Published:** 2022-03-18

**Authors:** Diego Oliva-Rico, Eunice Fabian-Morales, Rodrigo E. Cáceres-Gutiérrez, Adriana Gudiño, Fernanda Cisneros-Soberanis, Julieta Dominguez, Oscar Almaraz-Rojas, Cristian Arriaga-Canon, Clementina Castro-Hernández, Carlos De la Rosa, José L. Reyes, Luis A. Herrera

**Affiliations:** 1 Unidad de Investigación Biomédica en Cáncer, Instituto Nacional de Cancerología (INCan)—Instituto de Investigaciones Biomédicas, Universidad Nacional Autónoma de México (UNAM), Mexico City 14080, Mexico; andor.diego@gmail.com (D.O.-R.); fabeunice@gmail.com (E.F.-M.); rodrigocaceresgutierrez@gmail.com (R.E.C.-G.); areo28@hotmail.com (A.G.); juls.a.a@gmail.com (J.D.); carriaga.canon@gmail.com (C.A.-C.); ccastroh7@yahoo.com.mx (C.C.-H.); 2 Wellcome Centre for Cell Biology, Institute of Cell Biology (ICB), University of Edinburgh, Edinburgh EH9 3BF, UK; fda.cisneros@gmail.com; 3 Instituto Nacional de Medicina Genómica, Mexico City 14610, Mexico; oalmarazr@inmegen.gob.mx; 4 Departamento de Medicina Genómica y Toxicología Ambiental, Instituto de Investigaciones Biomédicas, Universidad Nacional Autónoma de México, Ciudad Universitaria, Mexico City 04510, Mexico; 5 Departamento de Biología Molecular de Plantas, Instituto de Biotecnología, Universidad Nacional Autónoma de México, Cuernavaca 62210, Mexico; cdlarosa@ibt.unam.mx (C.D.l.R.); jlreyes@ibt.unam.mx (J.L.R.)

**Keywords:** telomeres, TERRA, lncRNA, *hTERT*

## Abstract

The long noncoding RNA (lncRNA) telomeric repeat-containing RNA (TERRA) has been associated with telomeric homeostasis, telomerase recruitment, and the process of chromosome healing; nevertheless, the impact of this association has not been investigated during the carcinogenic process. Determining whether changes in TERRA expression are a cause or a consequence of cell transformation is a complex task because studies are usually carried out using either cancerous cells or tumor samples. To determine the role of this lncRNA in cellular aging and chromosome healing, we evaluated telomeric integrity and TERRA expression during the establishment of a clone of untransformed myeloid cells. We found that reduced expression of TERRA disturbed the telomeric homeostasis of certain loci, but the expression of the lncRNA was affected only when the methylation of subtelomeric bivalent chromatin domains was compromised. We conclude that the disruption in TERRA homeostasis is a consequence of cellular transformation and that changes in its expression profile can lead to telomeric and genomic instability.

## 1. Introduction

Telomeric instability has consistently been implicated in carcinogenesis. Several factors promote telomere integrity, including the shelterin complex, displacement/telomere loop (D/T-loop) formation, guanine quadruplex structures (G-quads), subtelomeric heterochromatin, and telomere length. These elements are associated with the regulation of abnormal cell division; however, evidence has shown that noncoding telomeric transcripts can also play a key role in the chromosome healing process, resulting in the protective mechanisms that promote telomeric homeostasis and thus prevent chromosome instability.

Telomeric repeat-containing RNA (TERRA) is one such transcript. This long noncoding RNA (lncRNA) binds telomeres via the shelterin complex [1] and assists in the maintenance of telomeric integrity by diverse mechanisms. TERRA can directly protect the telomeric sequence from degradation, forming DNA/RNA G-quad structures with single stranded DNA (ssDNA) that is displaced at the telomeres [2], and it preserves and renews the condensed state of subtelomeric chromatin via indirect interactions with chromatin-associated histone methyltransferases [3].

Although a definite mechanism between TERRA and telomere elongation has yet to be determined, different research groups have reported a variety of effects associated with TERRA expression that demonstrate a relationship between telomeric homeostasis and the regulated expression of this lncRNA [1,4,5,6,7,8,9,10]. For this reason, an abnormal TERRA expression profile is expected when telomeric stability is compromised. This phenomenon occurs in cancer, a disease in which cells transform by acquiring certain hallmarks during the course of the multistep process of tumorigenesis, which may lead to a malignant phenotype [4,5,6].

One of the many traits observed in cancerous cells is unlimited replication potential [11]. This excess replication is due to restoration of the telomeric sequences that are lost after each cell division, and TERRA participates in the regulation of this process. TERRA expression correlates with telomerase reverse transcriptase activity in cells that actively transcribe *hTERT*, the catalytic subunit of this enzyme, making TERRA-transcribing telomeres the most frequently elongated ones [12]. In *hTERT* null cells, TERRA expression is associated with accelerated telomere shortening [13], which primes telomeres for recombination in cells with alternative lengthening of telomeres (ALT).

This telomere-renewing phenotype, sustained either by telomerase expression or telomeric recombination, is essential for the continuation of a transformed cell lineage [11,14,15,16,17,18]. However, this process is only a facilitating hallmark in cell transformation [19]; the fundamental conditions that induce the loss of telomeric homeostasis remain vague. For this reason, studying the impact that altered transcription of TERRA has on the initiation of cancer could be important given that TERRA is critical for the telomeric integrity of both normal and transformed cells.

To study the role of TERRA in the process of cell transformation, we investigated its expression profile and how it changed during the process of clonal selection in untransformed cells. To this end, we evaluated TERRA expression in a multiclonal cell model that is prone to undergoing clonal selection during culture [20]. Given that altered expression of TERRA is ultimately reflected in telomeric stability, we monitored telomere length and subtelomeric chromatin compaction to associate any changes due to clone selection and cellular aging with fluctuations in the transcription of TERRA.

We selected the myelogenous lineage as our study model to take advantage of the stable telomeres that distinguish bone-marrow-derived neoplasias [21]. Since we intended to analyze telomeric stability, we compared the results obtained from expression analysis, telomere length measurement, and chromatin-mark enrichment between an untransformed cell line and a leukemia-derived cell line [22].

## 2. Results

### 2.1. Karyotype Analysis

**Figure 1:**
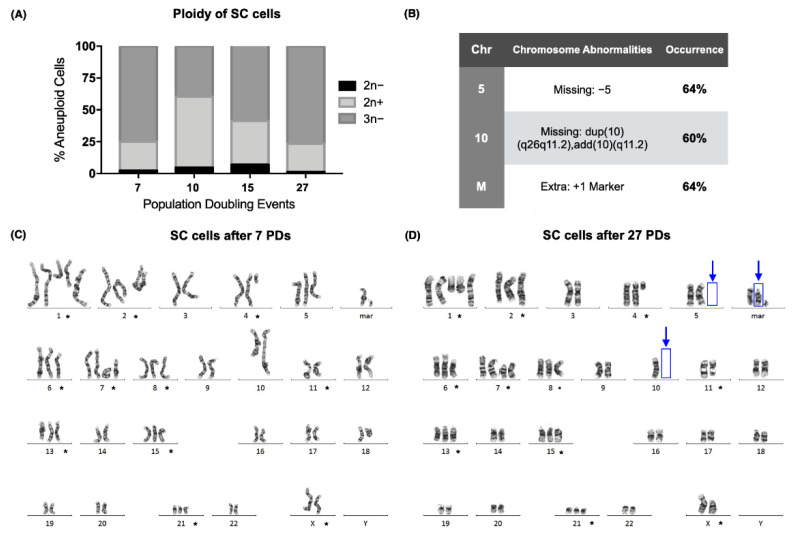
A hypotriploid clone of the SC cells was established, but numerical chromosomal instability was still present. (**A**) Percentage of aneuploid cells after 7, 10, 15, and 27 population-doubling events (PDs) of the SC cell line. Over 50% of the analyzed cells were hypotriploid (3n−) in 3 of the sampled PDs. (**B**) Table with the chromosomal abnormalities found after 27 PDs of the SC cell line. Approximately 60% of the analyzed cells displayed a loss of chromosome 5 and of a derivative chromosome 10, together with the appearance of an extra chromosome marker. (**C**) Karyogram of the SC cells after 7 population-doubling events. The numerical or structural chromosomal alterations that were found in every sampled population are marked (*). (**D**) Karyogram of the SC cells after 27 population-doubling events. The numerical or structural chromosomal alterations that were found in every sampled population are marked (*). Additional chromosomal abnormalities are marked with blue arrows.

Conventional karyotyping of the SC cell line (ATCC CRL-9855) proved that a clone was established in the early passages of the culture, with a modal number of 59, after only 7 population-doubling events (PDs). The modal number of the SC cells remained above 46 in each of the sampled passages, and the ploidy of the culture was hypotriploid in more than 50% of the analyzed metaphases during 3 of the evaluated cell passages (Figure 1A).

GTG-banding showed that the abnormal ploidy of the SC cell line was due to both numerical and structural abnormalities in its karyotype. Chromosomal alterations that were present on every doubling event are marked (*) (Figure 1C,D). Although a clone had already been established at the beginning of the assay, 3 new alterations were found after 27 PDs. We found that 60% of the analyzed metaphases were missing dup(10)(q26q11.2)add(10)(q11.2), and 64% were missing +5 and had acquired an extra marker chromosome (Figure 1B,D).

We report the karyotype of the SC cell line we worked with after 10 and 27 population-doubling events (PDs) as follows [23]:

7 PDs: 55~61,X,del(X)(p11.2),+der(1)t(1;?) (q21;?),+der(2)t(2;17)(q37;q21),+del(4)(q21),+5,del(6)(p21),del(7)(p10),+i(7)(p10),+8,dup(10)(q26q11.2)add(10)(q11.2),der(10)t(10;X)(p13;p11.4),del(11) (q23),+13,+15,+21,+2mar[cp25].

27 PDs: 60~61,X,del(X)(p11.2),+der(1)t(1;?) (q21;?),+der(2)t(2;17)(q37;q21),+del(4)(q12),+del(6)(p21),+del(7)(p10),+i(7)(p10),+8,−10,der(10)t(10;X)(p13;p11.4),del(11)(q23),+13,+15,+21,+3mar[cp25].

**Figure 2:**
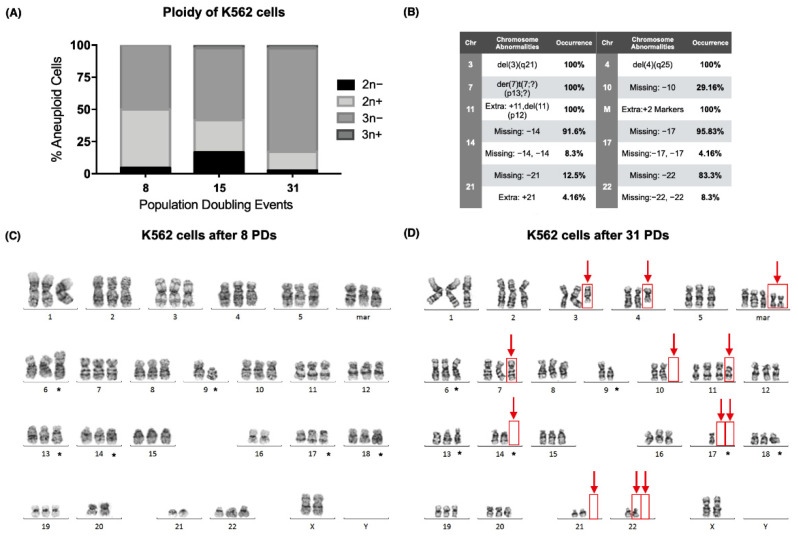
The hypotriploid K562 cell line displayed chromosomal instability as the culture aged. (**A**) Percentage of aneuploid cells after 8, 15, and 31 population-doubling events (PDs) in the K562 cell line. Over 50% of the analyzed cells were hypotriploid (3n−) in the sampled PDs. (**B**) Table with the chromosomal abnormalities found after 31 PDs of the K562 cell line. A total of 14 numerical/structural chromosomal alterations were found; 8 of them occurred in more than 80% of the analyzed cells. (**C**) Karyogram of the K562 cells after 8 population-doubling events. The numerical or structural chromosomal alterations that were found in every sampled population are marked (*). (**D**) Karyogram of K562 cells after 31 population-doubling events. The numerical or structural chromosomal alterations that were found in every sampled population are marked (*). Additional chromosomal abnormalities are marked with red arrows.

During our analysis, we found that the karyotype of the K562 cell line (ATCC CCL-243) also exhibited numerical and structural abnormalities that were not present at the time of their acquisition from the American Type Culture Collection (ATCC) (Figure 2A). Since the cell line is reported as hypotriploid, numerical abnormalities were expected, and stable chromosomal abnormalities were marked (*) (Figure 2C,D). However, several other abnormalities were found.

After 31 PDs, chromosomes 3 and 4 exhibited deletions, and a derivative chromosome 7 was found (der(7)t(7;?)(p13;?)), together with an additional chromosome 11 (+11,del(11)(p12)) and two extra marker chromosomes [23]. Furthermore, several chromosomes had lost 1 or 2 of their homologous pairs in some of the analyzed metaphases: loss of chromosome 10 (chr10) (29.16%), loss of chr14 (91.6%), loss of two chr14 (8.3%), loss of chr17 (95.8%), loss of two chr17 (4.16%), loss of chr21 (12.5%), loss of chr22 (83.3%), and loss of two chr22 (8.3%) (Figure 2B,D).

We report the karyotype of the K562 cell line we worked with after 8 and 31 population-doubling events (PDs) as follows [23]:

8 PDs: 61~74,XX,add(6)(p23),−9,del(9)(p13),der(13)t(13;?)(q14;?),−16,add(17)(p13.3),add(18)(q23),−20,−21,3mar[cp25].

31 PDs: 61~82,XX,del(3)(q21),del(4)(q25),add(6)(p23),der(7)t(7;?)(p13;?),−9,del(9)(p13),−10,+11,del(11)(p12),t(13;?)(q14;?),−14,−17,−17,add(18)(q23),−21,−22,5mar[cp25].

### 2.2. Telomerase Expression

According to the ATCC, SC was an undifferentiated, multiclonal cell line at the time of acquisition [20]; therefore, it was of interest for us to determine if telomerase expression was regained during our analysis. For this reason, we evaluated the expression of the catalytic subunit of telomerase, *hTERT*. We carried out this analysis in three cell lines: SC, a noncancerous myelogenous cell line; K562, a chronic myelogenous leukemia-derived cell line; and Saos2 (ATCC HTB-85), an osteosarcoma-derived cell line used as a negative control for the expression of the reverse transcriptase [24]. K562 cells served as our reference for *hTERT* expression [22]; therefore, the data were normalized against the earliest sampled population of the K562 cell line.

**Figure 3:**
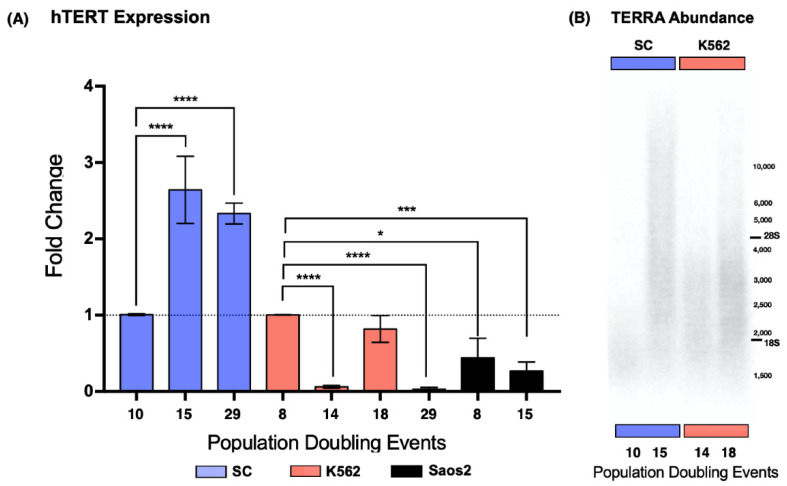
The expression of *hTERT* was recovered, and TERRA length increased in the SC cells. (**A**) Expression of *hTERT* in SC, K562, and Saos2 cells at different passages. *hTERT* showed increased and stable expression in SC cells. Fluctuating expression of *hTERT* was found in K562 cells. In Saos2 cells, *hTERT* expression remained significantly lower than that in myelogenous cell lines. Data were analyzed using ANOVA and Tukey’s multiple comparisons test. Adjusted *p* value < 0.0001 (****), =0.0006 (***), =0.0234 (*). (**B**) Northern blot analysis of TERRA abundance in the SC and K562 cell lines at different passages. There did not appear to be an increase in the global amount of TERRA in any of the sampled passages. However, there was an increase in the size of the lncRNA when *hTERT* expression was elevated in the SC cells (15 PDs) and in the K562 cells (18 PDs).

We found that the SC cell line displayed a 1.62-fold increase in the expression of *hTERT* after 15 PDs. After 29 PDs, *hTERT* expression was still elevated. In the K562 cell line, we found that the catalytic subunit expression was not consistent. After 14 PDs, *hTERT* expression dropped 0.93-fold, to almost undetectable levels, and 4 PDs later, *hTERT* expression returned to its original levels. Finally, after 29 PDs of the K562 cells, *hTERT* expression dropped 0.96-fold again. In contrast to the myeloid cell lines, *hTERT* expression remained below our reference levels in the ALT-dependent Saos2 cell line (Figure 3A). Since telomere maintenance is essential for unlimited replication, we did not expect *hTERT* expression to drop significantly in a leukemic cell line; nevertheless, this expression pattern could be a result of a successful malignant transformation, something that the recently established SC clone had yet to undergo.

### 2.3. Global TERRA Levels

We performed a Northern blot analysis using total RNA from both SC and K562 cells to determine if the changes in telomerase expression affected telomeric noncoding transcription at a global level. We chose to analyze the populations where the expression of *hTERT* had increased significantly.

Because we were working with a lncRNA bearing a repetitive sequence, we expected a smear of hybridized RNA. Our results showed hybridized telomeric RNA ranging from 1.5 kb to >10 kb. However, the bulk of TERRA was between 2.5 and 3.5 kb. In the SC cells, the bulk of the lncRNA was twice as long on the 15 PD samples (4 kb) than on the 10 PD samples (2 kb), and the maximum length of the blotted RNA was >10 kb after 15 PDs, a considerable increase compared to the 2.5 kb maximum length found after 10 PDs. Similar results were observed on the RNA spreads of K562 cells. The bulk of TERRA was longer on the 18 PD samples (3 kb) than on the RNA obtained after only 14 PDs (2.5 kb). The maximum length of the lncRNA also increased up to 6 kb in the samples from 18 PDs.

We found the shortest TERRA molecules after 10 PDs on the SC cells, with a maximum length of approximately 3 kb. In K562 cells, we found the shortest TERRA molecules after 14 PD events, with a maximum length of 4 kb. In both cell lines, the maximum length of this lncRNA increased to >10 kb after 5 PDs for SC, and up to 10 kb after 4 PDs for K562 (Figure 3B). Given that the length of TERRA increased when *hTERT* expression was elevated, it is safe to assume that the length of the telomeric track directly affects the size of the TERRAs transcribed from them. However, the transcription rate of this lncRNA must be regulated in a locus-specific way; otherwise, every length species of TERRA would accumulate proportionately.

### 2.4. Locus-Specific Expression of TERRA

Since we wanted to determine if the re-expression of *hTERT* could affect the expression of the telomeric lncRNA, we chose to evaluate a definite population of this lncRNA. Specifically, we examined the expression of TERRA 5p and TERRA 10q since these molecules are encoded in chromosomes where additional abnormalities were found after 27 PDs of the SC cell line.

Before the quantitative analysis of TERRA expression, we verified the coding potential of the selected loci. We used the coding potential calculator of the Center for Bioinformatics at Peking University in Beijing [25] to estimate the coding probability of the sequences we amplified [26,27]. In this analysis, we included published sequences from previously reported TERRA-expressing loci together with our sequences of interest.

The in silico analysis confirmed that the queried sequences had no open reading frame (ORF) integrity and that every sequence had a very low coding probability: 2.64 × 10^−3^ for TERRA 5p, 1.52 × 10^−6^ for TERRA 10q, 4.77 × 10^−7^ for TERRA 11q, 1.03 × 10^−2^ for TERRA 15q, and 0.114 for TERRA Xp (Figure S1).

**Figure 4:**
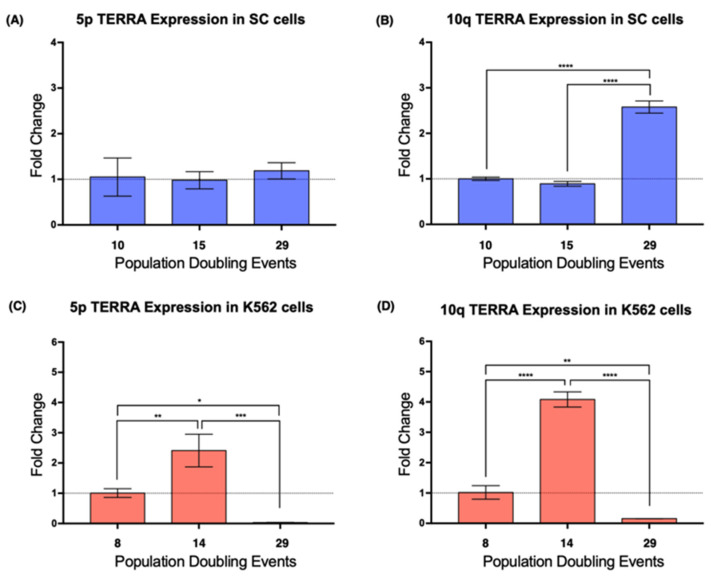
The expression of TERRA changed in a telomere-specific manner. (**A**) The expression of TERRA 5p was unaffected by cellular aging in SC cells. (**B**) Expression of TERRA 10q increased significantly after 29 PDs of the SC cells. (**C**,**D**) There was a similar expression pattern in both loci of the K562 cells. TERRA expression increased after 14 PDs and then dropped significantly after 29 PDs. Data were analyzed using ANOVA and Tukey’s multiple comparisons test. Adjusted *p* value < 0.0001 (****), =0.0003 (***), <0.005 (**), =0.0240 (*).

Real-time PCR results showed that, in the SC cells, TERRA 5p had stable expression during the assay, i.e., it was not affected by cellular aging, whereas the expression of TERRA 10q increased >2-fold after 29 PDs (Figure 4A,B). In K562 cells, the expression of TERRA 5p was stable on early passages, but after 29 PDs, its expression dropped significantly (Figure 4C). This abrupt decrease in expression was also found when we analyzed TERRA 10q after 29 PDs; however, TERRA 10q expression displayed a 4-fold increase after 14 PDs, a change that was not observed on TERRA 5p (Figure 4D). These results confirmed how TERRA expression varies depending not only on the age of the analyzed population, but also on the chromosome from where it was transcribed.

### 2.5. Global Telomeric Length

To study the relationship between TERRA expression and telomere integrity, we screened for fluctuations in telomere length during our analysis. Since we aimed to assess the *cis* effect of TERRA, we performed quantitative fluorescent in situ hybridization (Q-FISH) on metaphase spreads that were then arranged into karyograms for the specific identification of the telomeric signals from the short (p) and long (q) arms of each analyzed chromosome. We also analyzed the mean telomeric fluorescence intensity from every metaphase by comparing the sampled populations of each cell line to identify the global changes in telomere length from each cell line.

**Figure 5:**
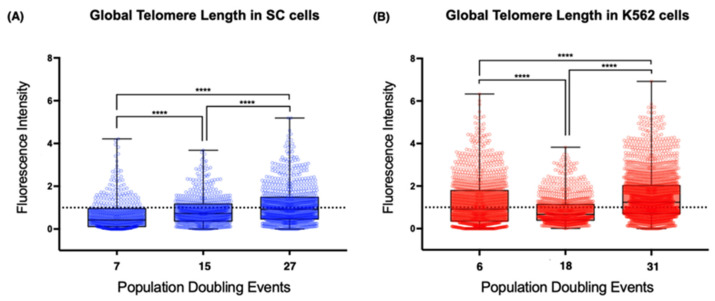
Global telomere length increased steadily in SC cells. (**A**) Dispersion of telomere length in different passages of the SC cell line. There was a discrete but steady lengthening of telomeric sequences during the assay. (**B**) Dispersion of telomere length in the K562 cell line at different passages. There was a significant reduction in telomere length after 18 PDs, but telomere length was recovered after 31 PDs. The dotted line in (**A**,**B**) represents the reference value used for the quantitative analysis of fluorescence intensity and the fluorescence from chromosome 18′s centromere. The fluorescence intensity of the telomeric probe was normalized against the centromeric fluorescence and expressed in arbitrary units (AU). Values > 1 represent an increase in hybridized telomeric sequences, i.e., lengthened telomeres. Values < 1 represent a loss of telemetric sequences, i.e., shortened telomeres. Data were analyzed using Kruskal–Wallis and Dunn’s multiple comparisons test. **** *p* < 0.0001.

Using the latter approach, we found evident differences between the telomeres of the non-neoplastic SC cells and those of the leukemia-derived K562 cells (Figure 5). First, the mean telomere length of the chromosomes from the earliest sampled populations was shorter on the SC cells (0.66 arbitrary units, AU) than the length on the chromosomes of the K562 cells (1.21 AU). The second difference was the loss of telomeric sequences after 18 PDs, when the mean fluorescence intensity was significantly decreased on K562 cells (0.855 AU). After 31 PDs, the intensity of the telomeric signal recovered its initial levels (1.5 AU). However, the mean fluorescence intensity of the SC cell telomeres increased steadily in each analyzed population. Finally, we found that, even though the telomere length had heterogeneous values in each of the analyzed cell populations, the difference was broader on K562 telomeres, whereas telomeres on the SC chromosomes had more discrete fluctuations in their length.

It appears that the re-expression of *hTERT* does not guarantee the efficient extension of every telomere. The effect of telomerase was clearly different between cell lines, but the presence of TERRA did not seem to affect this because, while TERRA’s global levels were high, the telomeres on the SC cells were extended (15 PDs), whereas the telomeres of the K562 cells shortened abruptly (18 PDs).

### 2.6. Chromosome-Specific Telomere Length Analysis

**Figure 6:**
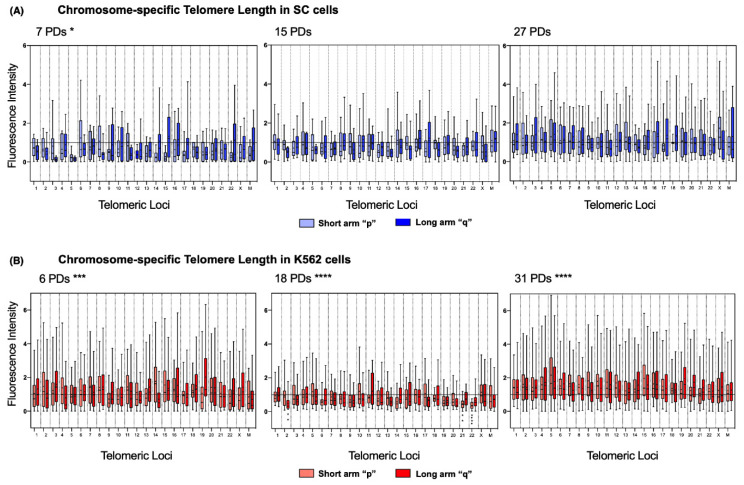
Chromosome-specific telomere length in SC and K562 cells (**A**) In the chromosome arms of the SC cells, we observed steady telomere lengthening. The initial telomere length was significantly heterogeneous at 7 PDs; several chromosome arms displayed critically short telomeres. In later passages, telomere length was recovered, and the dispersion of length values was no longer significant. Data were analyzed using Kruskal–Wallis and Dunn’s multiple comparisons tests. The *p* value for telomere length at 7 PDs was 0.043 (*). (**B**) In the chromosome arms of the K562 cells, we observed abrupt telomere shortening, followed by heterogeneous lengthening. The initial telomere length was significantly longer than that in the SC cells, but after 18 PDs, the telomere length decreased considerably. Telomeres 2q, 21p, and 22p were significantly shorter after 18 PDs in every analyzed cell. Adjusted *p* values for telomeres 2q, 21p, and 22p after 18 PDs < 0.005 (**) and <0.0001 (****). After 31 PDs, telomere length recovered, but the dispersion of length values remained statistically significant. Data were analyzed using Kruskal-Wallis and Dunn’s multiple comparisons tests. (**A**,**B**) The fluorescence intensity of the telomeric probe was normalized against a centromeric probe and expressed in arbitrary units (AU). The mean value is shown in every box (•). The *p* value for telomere length at 6 PDs was 0.0002 (***), and after 18 and 31 PDs, it was <0.0001 (****).

To address length heterogeneity in detail, we analyzed the telomeres of each chromosome arm individually using DAPI-based karyotyping. We found a significantly disparate telomere length across the chromosomes of the three sampled K562 populations (Figure 6B). In stark contrast, telomere length in SC chromosomes was only significantly different on the earliest sampled population of these cells (7 PDs). In later SC populations, the fluctuations in telomeric length were no longer significant (Figure 6A).

Detailed chromosome-specific analysis of the SC cells revealed that, after 7 PDs, 85.41% of the chromosome arms had short telomeres since their mean fluorescence intensity was below the reference value of the centromeric probe (1.0 AU). Furthermore, the median of the observed telomeric fluorescence was <1.0 AU on 95.83% of the chromosomes, and 52.17% of them were <0.5 AU. After 7 PDs, consistently short telomeres were found on 3q, 5p, 5q, 12p, 14p, 15p, and 22p of the SC cells. However, after 15 PDs, the intensity of telomeric fluorescence increased by 0.183 AU in every chromosome arm. After 27 PDs, only 31.25% of the telomeres in the SC cells had a mean fluorescence intensity <1.0 AU: 3p, 9q, 11q, 14p, 14q, 15p, 16p, 17p, 22p, and 22q. This finding indicates that telomeric sequences were recovered on 54.16% of the telomeres of the SC cells, with an increase of 0.251 AU to their mean length (Figure 6A).

The same analysis showed that, in the earliest sampled population (6 PDs) of K562 cells, only 27.08% of the chromosome arms had short telomeres: 4q, 9p, 10p, 12q, 14q, 22p, Xp, Mp, and Mq. Interestingly, most of the telomeres shortened after 18 PDs, with 79.16% of chromosome arms displaying a mean fluorescence <1.0 AU, and the median of the observed telomeric fluorescence was below the reference value on every chromosome arm. After 31 PDs, the fluorescence intensity values were again >1.0 AU in all the chromosome arms, which indicated that *hTERT* re-expression induced the recovery of telomeric sequences by 0.64 AU, almost doubling the amount lost after 18 PDs. By the end of the assay, telomeric sequences had recovered on almost 80% of the telomeres of the K562 cells (Figure 6B).

It is unclear whether the different rates in telomere extension could be related to the profile of *hTERT* expression, but it should be noted that an accelerated telomere elongation took place once the K562 cells regained *hTERT* expression, whereas in the SC cells, where *hTERT* was stably expressed, elongation was constant but more discrete.

### 2.7. Locus 5p and 10q-Specific Telomeric Length

Since our original intent was to study the relationship between TERRA expression and telomere length, we specifically analyzed the telomeres on chromosome arms 5p and 10q from each cell population.

**Figure 7:**
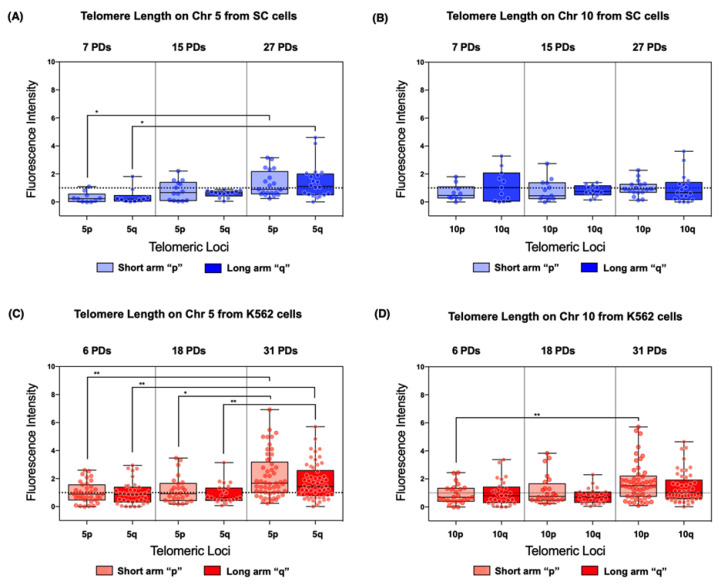
Individual chromosome arms displayed different lengthening patterns. Telomere length was evaluated on chromosomes 5 and 10. (**A**,**C**) In both cell lines, the telomeres from chromosome 5 were significantly extended after 27 PDs in SC cells and after 31 PDs in K562 cells. (**B**) No discernible lengthening occurred in chromosome 10 of the SC cells. (**D**) A lengthening pattern was evident in chromosome 10 of the K562 cells, but the change was only significant at locus 10p. (**A**–**D**) The fluorescence intensity of the telomeric probe was normalized against a centromeric probe and expressed in arbitrary units (AU). Data were analyzed with a Kruskal–Wallis test and Dunn’s multiple comparisons test. Adjusted *p* value < 0.05 (*), <0.005 (**).

On chromosome 5 of the SC cells, the mean telomere length of both arms displayed a significant increase after 27 PDs. Telomeres on 5p and 5q increased 3.0- and 2.615-fold in length, respectively, when compared to their initial length (Figure 7A). Although the mean telomere length increased in both sampled populations of the SC cell line, the difference was only significantly larger after 27 PDs. In contrast, on chromosome 10, the mean telomere length did not show significant changes across the sampled PDs (Figure 7B).

We found similar results in K562 cells. On chromosome 5, the mean telomere length of both arms displayed a statistically significant increase. Telomeres on 5p increased 1.319- and 1.137-fold after 18 and 31 PDs, respectively, compared to their initial length. Telomeres on 5q increased 1.022- and 0.854-fold after 18 and 31 PDs, respectively (Figure 7C). On chromosome 10, there was only a significant change in the mean telomere length of 10p, increasing 0.988-fold after 31 PDs (Figure 7D).

As previously noted, while identifying changes in telomere length, we observed heterogeneous values in each of the analyzed cell populations. Upon closer analysis, we found that global telomere length values had a broader range on the chromosomes of the K562 cell line compared with those of the SC cells (Figure 6). Across the analyzed populations of the SC cell line, we found more homogeneous telomere length values in both 5p and 10q than on the telomeres of the K562 cells. After 31 PDs of the K562 cells, the range of telomeric length on 5p and 5q increased 4.07 AU and 2.74 AU, respectively (Figure 7C). Moreover, the dispersion of telomeric length values in the SC cells only increased 1.81 AU on 5p and 2.79 AU on 5q (Figure 7A). This difference was also found on chromosome 10; after 31 PDs of the K562 cells, the range of telomeric lengths increased 3.16 AU on 10p and 1.25 AU on 10q (Figure 7D), whereas, in the SC cells after 27 PDs, the range only increased by 0.33 AU in the same chromosome arms (Figure 7B).

### 2.8. Subtelomeric Chromatin Analysis

The fluctuations we found in telomeric length can be due to the presence of telomerase; however, the telomere-binding proteins of the shelterin complex can hinder the activity of this enzyme at stable telomeres [28], thus explaining why shorter arms are preferentially elongated over others. Moreover, heterochromatin at any given subtelomeric locus can repress TERRA transcription [1], so there is a possible indirect link between telomere length, the state of the local chromatin and TERRA transcription in telomerase-expressing cells. Therefore, we decided to evaluate the enrichment of certain chromatin marks and associated proteins on the same loci where TERRA was quantified to link the aging-related changes in TERRA expression with the state of the subtelomeric chromatin.

#### 2.8.1. SC Cell Line—Heterochromatin

**Figure 8:**
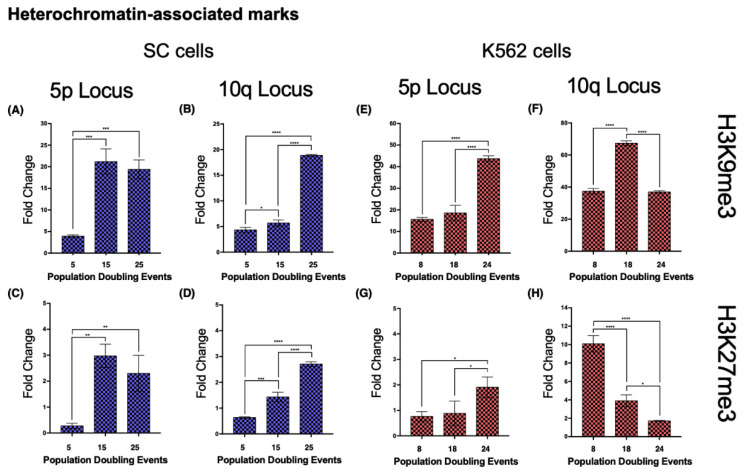
Heterochromatin-associated histone marks accumulated on both analyzed loci in the SC cell line. Chromatin immunoprecipitation was carried out to determine the abundance of the heterochromatin-associated histone marks, H3K9me3 and H3K27me3. Note the different scales on the axes of the graphs. (**A**–**D**) As the culture aged, both marks accumulated in loci 5p and 10q of the SC cells. (**E**,**G**) In K562 cells, locus 5p also accumulated both heterochromatin-associated marks. (**F**) The levels of H3K9me3 only increased temporarily at locus 10q after 18 PDs in K562 cells. (**H**) The levels of H3K27me3 decreased at locus 10q as K562 cells aged. Notably, in both cell lines, the levels of H3K9me3 were always considerably higher than those of H3K27me3. Data were analyzed using ANOVA and Tukey’s multiple comparisons test. Adjusted *p* value < 0.0001 (****), <0.001 (***), <0.01 (**), <0.05 (*).

**Figure 9:**
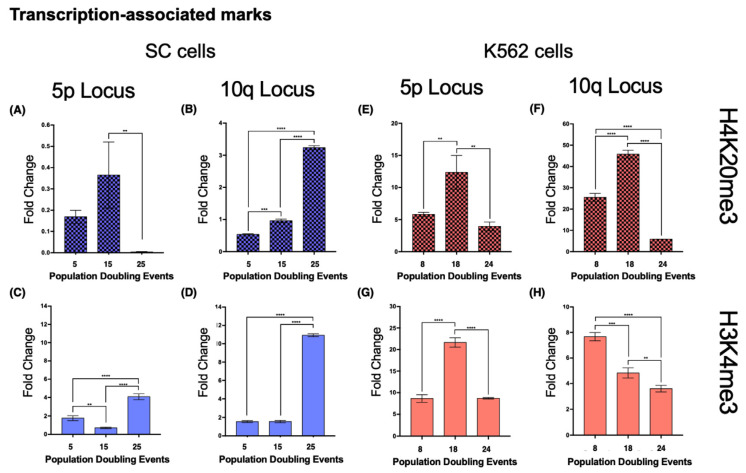
Antagonizing histone marks occurring in the same locus favored TERRA transcription. Chromatin immunoprecipitation was carried out to analyze the abundance of the histone marks, H4K20me3 and H3K4me3. Note the different scales on the axes of the graphs. (**A**) H4K20me3, a mark normally enriched in telomeric constitutive heterochromatin, had very low levels in locus 5p of the SC cells; after 25 PDs, this mark was nearly undetectable. (**B**) At locus 10q, the levels of H4K20me3 increased after 15 PDs and then became significantly enriched after 25 PDs. (**E**,**F**) The levels of H4K20me3 behaved similarly in both loci of the K562 cells; the mark accumulated after 18 PDs but then diminished after 24 PDs. The enrichment of H4K20me3 was considerably higher on K562 10q than on 5p. (**C**,**D**) H3K4me3, a mark associated with active gene promoters, accumulated in both loci of the SC cells after 25 PDs. The enrichment of H3K4me3 was higher on SC 10q than on 5p. (**G**) H3K4me3 also increased significantly after 18 PDs of the K562 cells, but the levels of the mark returned to their original value after 24 PDs. (**H**) The levels of H3K4me3 decreased gradually as the K562 cells aged. Data were analyzed using ANOVA and Tukey’s multiple comparisons test. Adjusted *p* value < 0.0001 (****), =0.0008 (***), <0.01 (**).

- Both loci 5p and 10q accumulated heterochromatin-associated histone marks as the cell population aged. In 5p, H3K9me3 increased 4.4-fold from its initial value after 15 PDs. The H3K27me3 mark increased 9.73-fold after 15 PDs and 7.29-fold after 25 PDs (Figure 8A,C). H4K20me3 was undetectable after 25 PDs (Figure 9A).
- In the 10q locus, H3K9me3 increased 3.32-fold only after 25 PDs. H3K27me3 displayed a slight but steady enrichment of 1.25-fold after 15 PDs, and 3.26-fold after 25 PDs (Figure 8B,D). H4K20me3 showed a 5-fold increase after 25 PDs (Figure 9B).

#### 2.8.2. K562 Cell Line—Heterochromatin

- When we analyzed the same chromatin marks in locus 5p of the K562 cell populations, we identified a similar phenomenon to that in the SC cells. Both H3K9me3 and H3K27me3 displayed a 1.8- and 1.49-fold enrichment, respectively, after 24 PDs (Figure 8E,G). H4K20me3 showed a 1.12-fold increase after 14 PDs, but after 24 PDs, the levels of this mark returned to their initial value (Figure 9E).
- At locus 10q, neither H3K9me3 nor H3K27me3 showed differences. H3K9me3 increased 0.79-fold after 14 PDs and then returned to its initial level after 24 PDs. The levels of H3K27me3 diminished 0.61-fold after 14 PDs and 0.83-fold after 24 PDs (Figure 8F,H). H4K20me3 displayed a 0.79-fold enrichment after 14 PDs, but the levels of this mark also decreased up to 0.76-fold below its initial levels after 24 PDs (Figure 9F).

#### 2.8.3. SC Cell Line—Euchromatin

**Figure 10:**
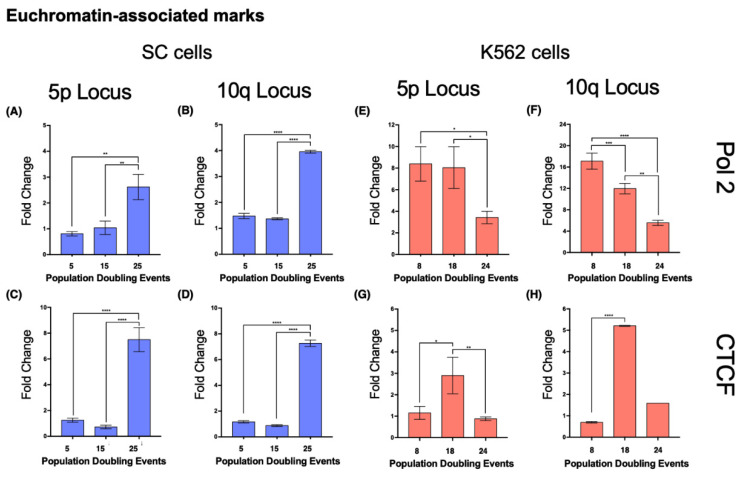
Euchromatin-associated proteins accumulated on both analyzed loci in the SC cell line. Chromatin immunoprecipitation was carried out to analyze the abundance of the euchromatin-associated proteins’ RNA Polymerase 2 (Pol 2) and the CCCTC binding factor (CTCF). Note the different scales on the axes of the graphs. (**A**–**D**) As the culture aged, both proteins accumulated in loci 5p and 10q of the SC cells. (**E**,**F**) The levels of Pol 2 diminished at loci 5p and 10q as the K562 cells aged. The reduced levels of Pol 2 that were found after 24 PDs in K562 cells were close to the levels accumulated on the same loci of the SC cells after 25 PDs. (**G**,**H**) The levels of CTCF increased on both loci after 18 PDs of the K562 cells, but after 24 PDs, they returned to their original values. Data were analyzed using ANOVA and Tukey’s multiple comparisons test. Adjusted *p* value < 0.0001 (****), = 0.0008 (***), < 0.01 (**), < 0.05 (*).

- In SC cells, euchromatin-associated marks accumulated in the 5p locus after 25 PDs, displaying a 2.228-fold increase in Pol II and a 4.98-fold increase in CTCF (Figure 10A,C). H3K4me3 also increased 1.316-fold after 25 PDs (Figure 9C).
- At locus 10q, there was a 1.67-fold increase in Pol II and a 5.21-fold increase in CTCF after 25 PDs (Figure 10B,D). The H3K4me3 mark increased 6.05-fold after 25 PDs (Figure 9D).

#### 2.8.4. K562 Cell Line—Euchromatin

- In the 5p locus of K562 cells, the levels of Pol II decreased 0.59-fold after 24 PDs. CTCF was enriched 1.5-fold after 14 PDs, but its levels returned to their initial value after 24 PDs (Figure 10E,G). H3K4me3 also increased 1.5-fold after 14 PDs, but after 24 PDs, the levels of this mark returned to their original value (Figure 9G).
- At locus 10q, the levels of Pol II decreased 0.3-fold after 14 PDs and 0.675-fold after 24 PDs. However, the levels of CTCF increased 6.48-fold after 14 PDs but then returned to their initial values after 24 PDs (Figure 10F,H). Finally, H3K4me3 diminished steadily 0.36-fold after 14 PDs and 0.53-fold after 24 PDs (Figure 9H).

## 3. Discussion

To determine the role of TERRA in cellular aging and chromosome healing, we evaluated chromosomal stability, telomere integrity, subtelomeric chromatin mark enrichment, and TERRA expression during the establishment of a clone of untransformed myeloid cells. Although we could not follow the process in which the hypotriploid SC clone was established, the cytogenetic analysis confirmed that we were working with a monoclonal cell line since the beginning of our assay [29]. This strategy in turn allowed us to assess the process of chromosome healing on a single clone as the culture aged. Since we were working with a cell line that underwent early spontaneous clonal selection [20], telomerase expression was expected [30,31,32], and we did find basal expression of this enzyme in SCs. However, there was an increase in *hTERT* expression after 15 PDs, and its levels remained higher than those we found in the reference leukemic cell line K562 [22]. Unlike in myeloid cells, in the ALT-dependent cell line Saos2, *hTERT* expression remained significantly lower since it does not depend on telomerase for the maintenance of its telomeres [33].

*hTERT* expression is crucial for leukemia progression [21,34,35,36,37], so it was of interest for us to find an increase in the transcription of reverse transcriptase in the SC samples. When reactivation of telomerase transcription occurs, most of the 5p region is either amplified or involved in chromosomal rearrangements that facilitate the transcription of *hTERT* [34,38,39,40]. However, Barthel et al. reported that 53% of the reactivation cases are due to the methylation of the promoter sequence of the *hTERT* gene, i.e., an epigenetic mechanism, which, in this particular case, promotes its transcription [22,41,42,43]. Since cytogenetic analysis of SC cells showed that the chromosome 5p region had not been compromised even after structural and numerical abnormalities had occurred, the reactivation of *hTERT* transcription in SC cells must be regulated by mechanisms unrelated to genetic alterations [44].

To support this idea, we examined *hTERT* expression in K562 cells because, in spite of the stable trisomic ploidy of chromosome 5 in both SC and K562 cells, we found a fluctuation in the expression of *hTERT* as the K562 cells aged. It appears that leukemic cells can reactivate *hTERT* expression in response to critical telomeric length and then silence its expression once telomeric homeostasis has been regained. This dynamic change in gene expression is more likely regulated by epigenetic factors, making histone post-translational modifications a better candidate to account for the expression changes caused by cellular aging [36,44]. The fluctuation in *hTERT* transcription could be responsible for the additional chromosomal alterations described above and for the indefinite dividing potential of the K562 cells, something that the recently established SC clone did not achieve. Moreover, it seems that the steady, elevated expression of *hTERT* induced cellular senescence in the SC cells, which did not divide past 29 PDs.

In *hTERT*-expressing cells, telomeric length is maintained within a set point by preventing telomerase from binding to longer telomeres and favoring its association with shortened sequences. This process is mediated by shelterin, the protein complex that binds and protects chromosome ends [28]. In our results, however, we found that, after critical telomeric length was addressed in K562 cells, the expression of *hTERT* was also downregulated. This result suggests a different regulatory mechanism that would induce another cycle of genomic instability and cell death but would also prevent senescence and establish cell immortality [36]. Without *hTERT*, the proliferation of K562 cells promoted telomere crisis, which drove the generation of further chromosomal rearrangements until *hTERT* was overexpressed again and the shortest telomeres were extended.

It appears that whenever *hTERT* expression increased, TERRA became longer. Interestingly, the maximum length of TERRA was >4 kb longer on the samples from the SC cells than on the leukemic K562 cells. Although we thought that the increased length of TERRA was an effect of the recovered telomerase activity, the results from the Saos2 cells showed that it is actually the length of the telomeric tract that influenced this association [45,46]. TERRA was longer and more abundant in ALT-dependent Saos2 cells (Figure S2) because of the increased association of RNA polymerase II at subtelomeric TERRA promoters, hypomethylation of their CpG islands, and reduced levels of the H3K9me3 mark [47]. Furthermore, in ALT-dependent cells, TERRA transcription promotes telomere recombination and thus telomeric elongation [48], which accounts for the higher abundance and length of the lncRNA that we found in Saos2 cells.

Despite the evident variations in the size of TERRA molecules in SC and K562 cells, the global abundance of TERRA was not affected by the re-expression of the reverse transcriptase. In the same way, neither the decreased telomeric transcription found in K562 cells nor the increased expression observed in either cell line had an impact on the overall levels of TERRA transcripts and was not detectable in the Northern blot.

We expected TERRA to have a more stable expression on the immortalized K562 cell line because it had already undergone accelerated telomere shortening and the consequent healing process that occurs during cell transformation [30,49,50,51,52]. However, it appears that the telomeres from the newly established SC clone were more stable. The analysis of telomere length showed that population doubling induced a statistically significant increase in the length of most telomeres from both myelogenous cell lines. Although elongation was not considerable, these results agree with what has been reported in hematological neoplasms, which can restore telomere function and maintain indefinite proliferation by expressing telomerase without elongating their telomeres in a substantial way [21,53].

In the presence of *hTERT*, population doubling induced a steady increase in the overall telomeric length of the SC cells. However, telomere length was significantly heterogeneous in every analyzed population of K562 cells, and even after a substantial loss of telomeric sequences, the heterogeneity in telomere length remained significant among the chromosome arms of K562 cells. After the noticeable attrition in the telomere length that occurred during PD 18 of the K562 cells, *hTERT* expression was recovered. These results indicate the series of events prompted by telomere shortening, in which re-expression of *hTERT* is a response induced when some telomeric sequences have reached a critical length [54]. The length differences found across the telomeres of every chromosome arm show that, although *hTERT* is present, telomere shortening results in a different response in each cell line.

Chromosome-specific analysis showed that, on the SC cells, most telomeres were adversely short at the beginning of our study, in stark contrast to the long telomeres of the K562 cells. The length disparities found across all telomeres in the SC cells were only significant at the earliest sampled population, which suggests that a steady telomere lengthening process occurred in the chromosome arms of the SC cells. In comparison, the telomeres of the K562 cell line did not have uniform growth; even after recovering from significant attrition, the length differences remained considerable across all telomeres in the K562 cells. However, it is worth mentioning that telomere recovery occurred at a higher rate in the leukemic-derived cells once *hTERT* expression was regained. The results show that critically short sequences were extended at a constant rate, gradually homogenizing the overall telomere length. However, in the event of abrupt telomere loss, accelerated recovery of telomeric sequences is induced to counteract sudden shortening.

Although the telomeres of both cell lines displayed changes in chromosomes 5 and 10, the lengthening trend was more evident in chromosome 5, whereas the increase in telomeric length of chromosome 10 was only detectable in the K562 cells. The significant elongation of chromosome 5 and not of chromosome 10 in the SC cells could be due to their initial length. The telomeres on chromosome 5 were among the shortest we found at the beginning of the assay; the initial telomeric length on chromosome 10 was similar to the mean length across all telomeres. In this way, the stable length of the telomeres on chromosome 10 can account for the lack of elongation since they have not yet undergone crucial attrition, and telomere extension occurs once a chromosome arm has reached a critical length [53]. In the case of K562 cells, telomeric length recovery was evident on both chromosomes 5 and 10. The elongation in chromosome 10 was only significant on the short arm (10p); however, the overall telomere length heterogeneity of this cell line could mask the significance of the changes observed on the long arm (10q).

In contrast to the SC cells, where the difference between the initial length of chromosomes 5 and 10 can explain the favored elongation of either of them, in the K562 cells, the average telomeric length in the earliest analyzed population was similar between chromosomes 5 and 10. In this case, the preferential elongation of chromosome 5 could not be critical length-induced, so we believe additional telomere homeostasis-maintaining mechanisms, other than telomerase, must be involved. Even if the analyzed telomeres were not significantly elongated, our results show that no further telomere shortening occurred when *hTERT* was present. In the case of the SC cells, where *hTERT* expression remained elevated as the cells aged, there was also a reduced occurrence of chromosomal abnormalities associated with telomere crisis, such as chromosome end-to-end fusion, nonreciprocal translocations, aneuploidy, and copy number change [36,55]. In contrast, we found 14 different chromosome abnormalities by the end of the assay in K562 cells, where *hTERT* expression had fluctuated significantly.

We compared the telomeric elongation reported by Q-FISH with the expression of TERRA at each locus to evaluate the cis effect that TERRA transcription could have on the telomeric length of a particular locus. Neither the unvarying lncRNA nor the overexpression of this lncRNA had a discernible impact on the average telomeric length at the evaluated loci. However, significant telomeric elongation was observed at the same locus where TERRA expression decreased to almost undetectable levels (Figure 4C,D and Figure 7C,D). We believe that there is an inversely proportional relationship between TERRA expression and the length of the telomere from which it is transcribed. This relation can be explained by the telomere position effect over long distances (TPE-OLD), where telomeres promote the spreading of heterochromatin in the subtelomeric region [56,57], and thus, telomere length affects the expression of TERRA instead of the reciprocal relationship.

Upon evaluation of heterochromatin-associated marks, we found distinct enrichment patterns depending on the analyzed subtelomeric locus. Both the constitutive heterochromatin mark H3K9me3 [58,59] and the facultative heterochromatin mark H3K27me3 [60] displayed a similar enrichment on locus 5p as the analyzed cell lines aged. Despite these increases, TERRA 5p transcription was only impaired in K562 cells, indicating that TERRA transcriptional regulation depends on a combination of factors that outweigh the abundance of either constitutive or facultative heterochromatin marks at its transcription start sites. A different enrichment pattern between cell lines was evident in 10q. As the SC cell line aged, there was an accumulation of both heterochromatin-associated marks on locus 10q. However, in the K562 cells, there was no increase in either mark at the same locus.

We included H4K20me3 in the analysis, a histone mark enriched at telomeric constitutive heterochromatin and reported to be involved both in the regulation of telomere elongation and the suppression of recombination [61]. Unlike the previous marks, the levels of H4K20me3 did not increase as the cells aged. In K562 cells, both evaluated loci displayed only a temporary increase in this telomeric heterochromatin mark. In the SC cells, the levels of H4K20me3 became nearly undetectable on the 5p locus by the end of the study; on the 10q locus of these cells, however, the levels of H4K20me3 had a 5-fold increase after 25 PDs.

Even though H3K9me3, H3K27me3, and H4K20me3 are all heterochromatin-associated histone marks and heterochromatinization is positively correlated with telomere length [56], H4K20me3 accumulated at the locus where no telomere elongation was observed: SC 10q (Figure 7B and Figure 10B). This inverse relationship between H4K20me3 enrichment and telomeric length has been reported when the expression of Suv4-20h1/h2 was abrogated, resulting in longer telomeres and more sister chromatid exchanges [62]. In this way, what favored telomere extension at the evaluated loci was the loss of histone methylation in subtelomeric heterochromatin and not just the transcription of TERRA or lack thereof. Notably, as the cultures aged, H3K27me3 accumulated in the evaluated loci of the SC cells. Since H3K27me3 is associated with facultative chromatin [60], its subtelomeric enrichment could be an indication that chromatin is poised for transcription. However, TERRA transcription increased in only one of the H3K27me3-enriched SC loci: 10q. For this reason, we believe that the transcription of this lncRNA is not regulated by the silencing complex responsible for the methylation of H3K27: PRC2 [63].

Since euchromatin allows access to the transcriptional machinery, in our analysis, we evaluated the enrichment of H3K4me3, a histone mark associated with active gene promoters [64], the abundance of the CCCTC-binding factor (CTCF), an insulator that prevents heterochromatin spreading and gene silencing [65,66,67], and the presence of serine 5-phosphorylated RNA polymerase 2, the enzyme responsible for TERRA transcription [68,69]. We found a distinct difference between the SC cells and the K562 cells upon analyzing euchromatin marks. In the latter, transcription-associated marks displayed either an unsustained increase or a steady reduction in their levels. Moreover, in the SC cells, euchromatin marks became enriched at both loci once 25 PDs had passed.

Although the most evident change in SC cells was the enrichment of H3K4me3 and the insulator CTCF, both analyzed loci displayed an accumulation of serine 5-phosphorylated RNA polymerase 2. Regardless of the poised chromatin state of the subtelomeric chromatin, TERRA transcription only increased on the SC 10q locus. The different expression rates between SC 5p and 10q could arise from the 6-fold enrichment of H3K4me3 on 10q, but the loss of H4K20me3 on the 5p locus is also a central part of this phenomenon. Bivalent chromatin domains enriched with both H3K4me3 and H4K20me3 are not only located near the transcriptional start sites (TSSs) of active genes but also display more efficient transcription since the domains marked with both of these histone modifications report less stalling of RNA polymerase 2 [70].

We believe that TERRA 10q transcription increased in SC cells as a result of a higher abundance of both H4K20me2 and H3K4me3, rather than just as a response to the presence of RNA polymerase. The concurrent presence of both histone marks also favored the transcription of TERRA 10q after 14 PDs of the K562 cells. Nevertheless, a reduction of polymerase II in both K562 loci was enough to impair TERRA transcription in later PDs.

Apart from the direct effect that the chromatin can have on the expression of TERRA, the epigenetic changes that were evidenced in our results could further impact telomere homeostasis at a structural level. It is well known how the spatial localization of the chromatin can impact genome stability and gene regulation [71]; in the same way, chromatin folding and chromatin domain integrity can impact the epigenome by maintaining the topological homeostasis of a cell [72]. Cancer, however, is characterized by altered gene expression, genome instability and epigenetic deregulation; these interconnected changes are associated with a robust alteration of the three-dimensional (3D) chromosomal organization that takes place during the initiation and progression stages of carcinogenesis [72,73,74].

The telomeres are no exception to spatial regulation. They behave as dynamic structures that stabilize chromosome positions within the nucleus, thus regulating transcriptional processes [73]. Telomeres have been described at the nuclear periphery [75], in association with the nuclear matrix [76], and throughout the entire nucleus [77], but it is clear that their usual 3D organization is altered when cancer develops [78,79,80,81].

This leads us to consider that the differences in telomere length we report can also be due to structural alteration ensued by changes in subtelomeric chromatin methylation. An abnormally stable association between telomeres and the nuclear lamina, brought about by an increase in repressive histone modifications such as H3K9me2/3, H3K27me3, and H4K20me2/3 [82] can lead to stalled replication forks, their collapse, and eventually to telomere shortening. Though quite possible, in order to determine the contribution of these topological features in the recovery of telomeric homeostasis, further studies are required to evaluate the association between the analyzed loci and the nuclear lamina.

## 4. Materials and Methods

### 4.1. Cell Culture

The osteosarcoma-derived cell line Saos2 and the myelogenous cell lines K562 and SC were all acquired from the American Type Culture Collection (ATCC HTB-85; ATCC CCL-243; ATCC CRL-9855, Manassas, VA, USA). K562 and SC cells were cultured in Iscove’s modified Dulbecco’s medium (IMDM) (ATCC 30-2005, Manassas, VA, USA) containing 10% fetal bovine serum (FBS) (ATCC 30-2020, Manassas, VA, USA). Additionally, the IMDM for the SC cells was supplemented with 0.05 mM 2-mercaptoethanol (Gibco 31350010, Waltham, MA, USA), 0.1 mM hypoxanthine, and 0.016 mM thymidine (hypoxanthine—thymidine (H.T.) 500× concentrate (ATCC 71-X, Manassas, VA, USA). Saos2 cells were cultured in McCoy’s 5a Medium (ATCC 30-2007, Manassas, VA, USA) containing 10% fetal bovine serum (FBS) (ATCC 30-2020, Manassas, VA, USA). All the cells were maintained at 37 °C in 5% CO_2_. Cells were subcultured whenever the culture reached 80% confluence. Cell populations were sampled every 3–4 passages.

### 4.2. Metaphase Preparation

The cells were incubated for 15 min at 37 °C in their corresponding growth medium supplemented with Colcemid (Gibco 15212012, Waltham, MA, USA) to a final concentration of 100 ng/mL. The suspended K562 and SC cells were washed with PBS and resuspended in a 0.55% potassium chloride hypotonic solution. The adherent Saos2 cells were rinsed with PBS and then added to a 0.8% sodium citrate hypotonic solution. The cells were incubated for 30 min at 37 °C and then prefixed by supplementing the medium with 1 mL of a fixative solution that contained 3 parts methanol and 1 part acetic acid for the suspended cells, and 5 parts methanol and 2 parts acetic acid for the adherent cells. The cells were then centrifuged and resuspended in their corresponding fixative solutions for 5 min at room temperature. Slides were prepared with the cell suspension.

### 4.3. Conventional Karyotype Analysis

Metaphase spreads were stained with Giemsa (Merck Millipore 1.09204.0500, Burlington, MA, USA) for chromosome counting. In the SC cell line, we counted 44, 43, 29, and 81 metaphases to determine the line’s ploidy after 7, 10, 15, and 27 PDs, respectively. For the K562 cell line ploidy analysis, we counted 25, 106, and 43 metaphases after 8, 18, and 31 PDs, respectively. Slides were also processed for GTG banding of both cell lines. Twenty-five metaphases were analyzed by conventional karyotyping for both the SC and K562 cell lines. The PDs analyzed were 7 and 27 on SC cells and 8 and 31 on K562 cells.

### 4.4. Northern Blot

Twenty micrograms of total RNA from each condition was used for Northern blot analysis using a NorthernMax^®^ Kit (Applied Biosystems AM1940, Waltham, MA, USA) according to the manufacturer’s instructions. RNA samples were size-fractionated by electrophoresis in a denaturing 1.4% agarose/is% formaldehyde gel. RNA was transferred to a Hybond-N+ nylon membrane (Amersham Hybond, RPN3050B, GE Healthcare, Chicago, IL, USA) and hybridized with dCTP[P32]-labeled DNA probes specific to the telomeric sequence.

### 4.5. Primer Design

The subtelomeric loci chosen to analyze TERRA transcription and chromatin mark enrichment have already been described as TERRA-transcribing loci [3,7]. The primers for loci 5p, 11q, and Xp were designed to amplify subtelomeric regions with DNase-sensitive sites, reported enhancer sequences, predicted transcription start sites (TSS), and enriched with chromatin marks such as H3K4me3, H3K4me1, and Pol II. We used the University of California Santa Cruz’s Genome Browser to carry out this analysis [83,84]. Loci 15q and 10q were analyzed using the primers reported in [7].

### 4.6. Analysis of hTERT and TERRA Expression

Total RNA was isolated from each cell line at different passages using the Direct-zol RNA Miniprep kit (Zymo Research, R2050, Irvine, CA, USA). Two micrograms of RNA was used for cDNA synthesis using the High-Capacity cDNA Reverse Transcription kit (Thermo Fisher, 4368814, Waltham, MA, USA). qPCR was carried out on a StepOne Real-Time PCR system using the Maxima SYBR Green/ROX qPCR Master Mix (Thermo Fisher, K0221). The primers used for TERRA quantification were previously reported by Deng et al. [3,7] as TERRA-expressing loci; the 5p set of primers was designed to amplify a locus of potential expression of this lncRNA. The fold change in TERRA expression was calculated using delta delta Ct, taking the earliest population-doubling event as our reference. All values for *hTERT* expression analysis were normalized against GAPDH. Values for TERRA analysis were normalized against U6 using primers reported by Galiveti et al. [85].

### 4.7. Q-FISH for Telomere Length Analysis

Q-FISH was performed on metaphases from at least three population-doubling events of the K562 and SC cell lines. A pan-telomeric FITC-labeled probe was used for telomere length quantification (DAKO, K532511, Glostrup, Denmark). A Cy5-labeled chromosome 18 centromeric probe (MetaSystems, D-0818-050-FI, Altlussheim, Germany) was used as a fluorescence intensity reference for telomere length analysis. The probes were hybridized sequentially, preparing the slides first for the telomeric probe and then for the centromeric reference. The slides were permeabilized by incubating in a 0.005% pepsin, 0.01 M HCl solution for 1 min at 37 °C, and then washed twice in TBS before denaturing using a Hybrite Slide Stainer (Abbott Molecular, Des Plaines, IL, USA) for 5 min at 80 °C for the telomeric probe and 5 min at 75 °C for the centromeric probe. The manufacturer’s protocol was subsequently followed (Dako, K532511, Glostrup, Denmark).

### 4.8. Image Processing

Metaphases were captured using an Axio Imager Z2 microscope (Carl Zeiss, GmbH, Jena, Germany) coupled with a Metafer module for automatic metaphase search and capture (MetaSystems Hard und Software, GmbH, Altlussheim, Germany). High-quality hybridized metaphases were scanned with a Plan-APOCHROMAT 63x/1.4 Oil objective and three optical filters for the detection of DAPI, FITC, and Cy5 (Carl Zeiss, GmbH). The resulting images were then processed with Ikaros software for conventional karyotype analysis and with Isis software for DAPI-based karyotyping and telomere length quantification (MetaSystems Hard und Software, GmbH, Altlussheim, Germany).

The telomere length of individual chromosome arms was calculated based on the fluorescence intensity of the hybridized probes on the telomeric repeat. We used the fluorescence of chromosome 18′s centromere (Chr18) as a reference value, normalizing the telomeric fluorescence intensity of each chromosome arm against the hybridized centromere of each analyzed metaphase. The fluorescence intensity of the telomeric probe was normalized against the centromeric fluorescence and expressed in arbitrary units (AU). In this way, fluorescence intensity values of >1.0 correspond to an increase in the amount of hybridized telomeric probes and were interpreted as lengthened telomeres. A fluorescence intensity of <1.0 corresponds to a decrease in the amount of hybridized telomeric probes and was interpreted as a partial loss of telomeric sequences, i.e., shortened telomeres.

### 4.9. Chromatin Immunoprecipitation

Chromatin was isolated and immunoprecipitated using the Shearing ChIP Kit (Diagenode, C01020012, Denville, NJ, USA) and the OneDay ChIP kit (Diagenode, C01010080, Denville, NJ, USA), respectively, according to the manufacturer’s protocol. To immunoprecipitate H3K4me3 (Abcam, ab8580, Waltham, MA, USA), H3K9me3 (Abcam, ab8898, Waltham, MA, USA), H3K27me3 (Millipore, 07449, Darmstadt, Germany), H4K20me3 (Abcam, ab9053, Waltham, MA, USA), CTCF (Millipore, 07729, Darmstadt, Germany), and RNA Pol-II (Abcam, ab5408), we used 4 µg of each antibody and incubated them overnight. Immunoprecipitated DNA was analyzed by qPCR with the StepOne Real-time PCR system (Thermo Fisher Scientific, 4376600, Waltham, MA, USA) using specific primers for each locus. Total chromatin input from every sample was used as a reference for the comparative curves of the analyzed marks. Data were normalized against IgG.

## 5. Conclusions

Our study showed how a cellular clone regained telomeric homeostasis by recovering telomerase expression after undergoing structural and numerical abnormalities. Increased telomerase transcription was found to hinder further chromosome abnormalities in the established clone of the SC cell line. In K562 cells, in which telomerase expression was not stable, further chromosomal abnormalities occurred.

Despite the apparently regained homeostasis, telomeres in SC displayed constant lengthening; however, SC cells stopped dividing even in the presence of telomerase. Therefore, *hTERT* re-expression and telomeric recovery were not enough to transform human myelogenous cells; further alterations, such as HRAS, TP53, and RB1 mutations [86,87,88], must occur as the cells age so as to induce cell transformation and to attain the hallmark unlimited replication potential of cancerous cells.

There is a correlation between the recovery of telomerase expression and a considerable increase in the length of TERRA. It should be noted that this is directly related to telomere length, so TERRA length can increase in the absence of *hTERT*.

We found that an abrupt reduction in TERRA expression preceded an accelerated elongation of the telomeres from either locus of the K562 cells; therefore, the homeostasis of the analyzed telomeres did have an in cis relation with the lncRNA transcribed from its adjacent subtelomeric region. However, the interplay between telomeric transcription and telomere stability appeared to depend more on histone methylation at the associated subtelomeric region than a direct effect from TERRA transcription. This finding was evidenced by the effect of the loss of H4K20me3, which impaired TERRA transcription in euchromatin mark-enriched loci that were otherwise poised for transcription.

This result leads us to believe that, in the process of chromosome healing that follows telomere crisis and genomic instability, the recovery of heterochromatin at subtelomeres favors the stabilization of telomeres, not just the presence of telomerase. By first accumulating H4K20me3/H3K4me3 and promoting TERRA transcription, critically short telomeres recruit telomerase. Once telomere elongation has begun, the heterochromatin marks H3K27me3/H3K9me3 accumulate in the adjacent subtelomeric region and limit TERRA expression. This process stabilizes telomere length within a set point between replication-induced shortening and telomerase-mediated lengthening.

**Figure 11:**
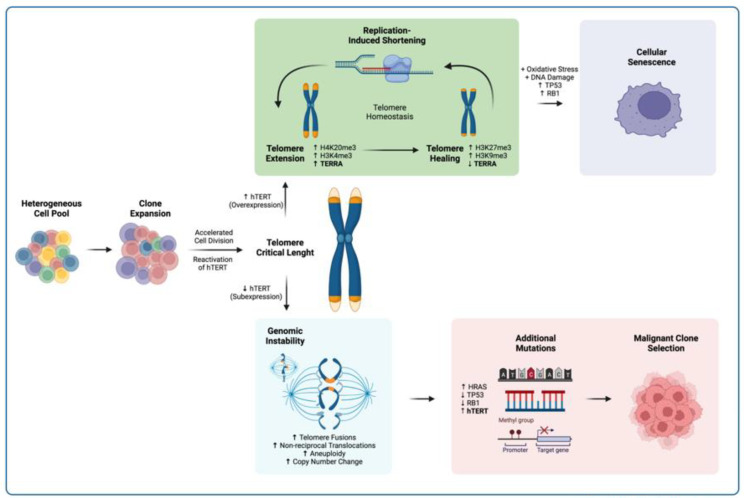
Proposed model for TERRA involvement in telomere recovery. In spite of *hTERT* reactivation, the expansion of a cell clone leads to critical telomere length due to an accelerated cell division. If *hTERT* is overexpressed, telomere healing ensues, and homeostasis is recovered. If *hTERT* expression does not resolve telomere loss, then genomic instability takes place. If the conditions are met, both scenarios can further develop. Upper panel. In cells with stable telomere length, proliferation can still be halted. Accumulation of DNA damage under physiological levels of oxidative stress can lead to the TP53/RB1-mediated cell cycle arrest and the induction of cellular senescence [18,89]. Lower panel. In cells with critical telomere length, further mutations can accumulate due to genomic instability. Mutations in genes such as HRAS, TP53, and RB1, together with the re-expression of *hTERT*, can prompt a cell towards malignant transformation [86,87,88].

In this way, reduced expression of TERRA can contribute to telomeric instability and prompt the development of chromosomal abnormalities as cells divide in the absence of stable telomeres. Thus, a disruption in TERRA homeostasis is not a cause but a consequence of cellular transformation, and changes in its expression profile can lead to telomeric and genomic instability that can either initiate premature cellular senescence or enable the selection of a cellular clone with unlimited replication potential (Figure 11).

## Data Availability

Not applicable.

## Supplementary Materials

The following supporting information can be downloaded at https://www.mdpi.com/article/10.3390/ijms23063271/s1.

## References

[1] Deng Z., Norseen J., Wiedmer A., Riethman H., Lieberman P.M. (2009). TERRA RNA Binding to TRF2 Facilitates Heterochromatin Formation and ORC Recruitment at Telomeres. Mol. Cell.

[2] Griffith J.D., Comeau L., Rosenfield S., Stansel R.M., Bianchi A., Moss H., Lange T. (1999). de Mammalian Telomeres End in a Large Duplex Loop. Cell.

[3] Arnoult N., Beneden A.V., Decottignies A. (2012). Telomere Length Regulates TERRA Levels through Increased Trimethylation of Telomeric H3K9 and HP1α. Nat. Struct. Mol. Biol..

[4] Oliva-Rico D., Herrera L.A. (2017). Regulated Expression of the LncRNA TERRA and Its Impact on Telomere Biology. Mech. Ageing Dev..

[5] Flynn R.L., Centore R.C., O’Sullivan R.J., Rai R., Tse A., Songyang Z., Chang S., Karlseder J., Zou L. (2012). TERRA and HnRNPA1 Orchestrate an RPA-to-POT1 Switch on Telomeric Single-Stranded DNA. Nature.

[6] Xu Y., Komiyama M. (2012). Structure, Function and Targeting of Human Telomere RNA. Methods.

[7] Deng Z., Wang Z., Stong N., Plasschaert R., Moczan A., Chen H.-S., Hu S., Wikramasinghe P., Davuluri R.V., Bartolomei M.S. (2012). A Role for CTCF and Cohesin in Subtelomere Chromatin Organization, TERRA Transcription, and Telomere End Protection. EMBO J..

[8] Thijssen P.E., Tobi E.W., Balog J., Schouten S.G., Kremer D., Bouazzaoui F.E., Henneman P., Putter H., Slagboom P.E., Heijmans B.T. (2013). Chromatin remodeling of human subtelomeres and TERRA promoters upon cellular senescence: Commonalities and differences between chromosomes. Epigenetics.

[9] Beishline K., Vladimirova O., Tutton S., Wang Z., Deng Z., Lieberman P.M. (2018). CTCF Driven TERRA Transcription Facilitates Completion of Telomere DNA Replication. Nat. Commun..

[10] Mei Y., Deng Z., Vladimirova O., Gulve N., Johnson F.B., Drosopoulos W.C., Schildkraut C.L., Lieberman P.M. (2021). TERRA G-Quadruplex RNA Interaction with TRF2 GAR Domain Is Required for Telomere Integrity. Sci. Rep..

[11] Hanahan D., Weinberg R.A. (2000). The Hallmarks of Cancer. Cell.

[12] Farnung B.O., Brun C.M., Arora R., Lorenzi L.E., Azzalin C.M. (2012). Telomerase Efficiently Elongates Highly Transcribing Telomeres in Human Cancer Cells. PLoS ONE.

[13] Arora R., Lee Y., Wischnewski H., Brun C.M., Schwarz T., Azzalin C.M. (2014). RNaseH1 Regulates TERRA-Telomeric DNA Hybrids and Telomere Maintenance in ALT Tumour Cells. Nat. Commun..

[14] Gutschner T., Diederichs S. (2012). The Hallmarks of Cancer. RNA Biol..

[15] Bryan T.M., Englezou A., Gupta J., Bacchetti S., Reddel R.R. (1995). Telomere Elongation in Immortal Human Cells without Detectable Telomerase Activity. EMBO J..

[16] Shay J.W., Bacchetti S. (1997). A Survey of Telomerase Activity in Human Cancer. Eur. J. Cancer.

[17] Shay J.W., Wright W.E. (2000). Hayflick, His Limit, and Cellular Ageing. Nat. Rev. Mol. Cell Biol..

[18] Collado M., Blasco M.A., Serrano M. (2007). Cellular Senescence in Cancer and Aging. Cell.

[19] Hanahan D., Weinberg R.A. (2011). Hallmarks of Cancer: The Next Generation. Cell.

[20] Collins G.W., Largen M.T. (1995). Continuous Mammalian Cell Lines Having Monocyte/Macrophage Characteristics and Their Establishment In Vitro. U.S. Patent.

[21] Nogueira B.M.D., Machado C.B., Montenegro R.C., Moraes M.E.A.D., Moreira-Nunes C.A. (2020). Telomere Length and Hematological Disorders: A Review. In Vivo.

[22] Zinn R.L., Pruitt K., Eguchi S., Baylin S.B., Herman J.G. (2007). *hTERT* Is Expressed in Cancer Cell Lines Despite Promoter DNA Methylation by Preservation of Unmethylated DNA and Active Chromatin around the Transcription Start Site. Cancer Res..

[23] McGowan-Jordan J., Hastings R.J., Moore S. (2020). ISCN 2020: An International System for Human Cytogenomic Nomenclature.

[24] Scheel C., Schaefer K.-L., Jauch A., Keller M., Wai D., Brinkschmidt C., van Valen F., Boecker W., Dockhorn-Dworniczak B., Poremba C. (2001). Alternative Lengthening of Telomeres Is Associated with Chromosomal Instability in Osteosarcomas. Oncogene.

[25] Coding Potential Calculator. http://cpc2.gao-lab.org/index.php.

[26] Kang Y.-J., Yang D.-C., Kong L., Hou M., Meng Y.-Q., Wei L., Gao G. (2017). CPC2: A Fast and Accurate Coding Potential Calculator Based on Sequence Intrinsic Features. Nucleic Acids Res..

[27] Kong L., Zhang Y., Ye Z.-Q., Liu X.-Q., Zhao S.-Q., Wei L., Gao G. (2007). CPC: Assess the Protein-Coding Potential of Transcripts Using Sequence Features and Support Vector Machine. Nucleic Acids Res..

[28] Lange T. (2005). de Shelterin: The Protein Complex That Shapes and Safeguards Human Telomeres. Gene Dev..

[29] Jones N. (2002). Human Cytogenetics: Malignancy and Acquired Abnormalities, Third Edition, A Practical Approach. Chromosome Res..

[30] Halvorsen T.L., Leibowitz G., Levine F. (1999). Telomerase Activity Is Sufficient To Allow Transformed Cells To Escape from Crisis. Mol. Cell Biol..

[31] Kamal S., Junaid M., Ejaz A., Bibi I., Akash M.S.H., Rehman K. (2020). The Secrets of Telomerase: Retrospective Analysis and Future Prospects. Life Sci..

[32] Lewis K.A., Tollefsbol T.O. (2016). Regulation of the Telomerase Reverse Transcriptase Subunit through Epigenetic Mechanisms. Front. Genet..

[33] Giardini M.A., Segatto M., da Silva M.S., Nunes V.S., Cano M.I.N. (2014). Telomere and Telomerase Biology. Telomeres in Health and Disease.

[34] Ropio J., Merlio J.-P., Soares P., Chevret E. (2016). Telomerase Activation in Hematological Malignancies. Genes.

[35] Vicente-Dueñas C., Barajas-Diego M., Romero-Camarero I., González-Herrero I., Flores T., Sánchez-García I. (2012). Essential Role for Telomerase in Chronic Myeloid Leukemia Induced by BCR-ABL in Mice. Oncotarget.

[36] Roake C.M., Artandi S.E. (2020). Regulation of Human Telomerase in Homeostasis and Disease. Nat. Rev. Mol. Cell Biol..

[37] Deville L., Hillion J., Ségal-Bendirdjian E. (2009). Telomerase Regulation in Hematological Cancers: A Matter of Stemness?. Biochim. Biophys. Acta Mol. Basis Dis..

[38] Zhao Y., Wang S., Popova E.Y., Grigoryev S.A., Zhu J. (2009). Rearrangement of Upstream Sequences of the *hTERT* Gene during Cellular Immortalization. Genes Chromosomes Cancer.

[39] Zhang A., Zheng C., Lindvall C., Hou M., Ekedahl J., Lewensohn R., Yan Z., Yang X., Henriksson M., Blennow E. (2000). Frequent Amplification of the Telomerase Reverse Transcriptase Gene in Human Tumors. Cancer Res..

[40] Heidenreich B., Kumar R. (2017). TERT Promoter Mutations in Telomere Biology. Mutat. Res. Rev..

[41] Barthel F.P., Wei W., Tang M., Martinez-Ledesma E., Hu X., Amin S.B., Akdemir K.C., Seth S., Song X., Wang Q. (2017). Systematic Analysis of Telomere Length and Somatic Alterations in 31 Cancer Types. Nat. Genet..

[42] Greider C.W. (2006). Telomerase RNA Levels Limit the Telomere Length Equilibrium. Cold Spring Harb. Sym..

[43] Cristofari G., Lingner J. (2006). Telomere Length Homeostasis Requires That Telomerase Levels Are Limiting. EMBO J..

[44] Zhu J., Zhao Y., Wang S. (2010). Chromatin and Epigenetic Regulation of the Telomerase Reverse Transcriptase Gene. Protein Cell.

[45] Episkopou H., Draskovic I., Beneden A.V., Tilman G., Mattiussi M., Gobin M., Arnoult N., Londoño-Vallejo A., Decottignies A. (2014). Alternative Lengthening of Telomeres Is Characterized by Reduced Compaction of Telomeric Chromatin. Nucleic Acids Res..

[46] Beneden A.V., Arnoult N., Decottignies A. (2013). Telomeric RNA Expression: Length Matters. Front. Oncol..

[47] Arora R., Azzalin C.M. (2015). Telomere Elongation Chooses TERRA ALTernatives. RNA Biol..

[48] Silva B., Arora R., Bione S., Azzalin C.M. (2021). TERRA Transcription Destabilizes Telomere Integrity to Initiate Break-Induced Replication in Human ALT Cells. Nat. Commun..

[49] Véronèse L., Tournilhac O., Callanan M., Prie N., Kwiatkowski F., Combes P., Chauvet M., Davi F., Gouas L., Verrelle P. (2013). Telomeres and Chromosomal Instability in Chronic Lymphocytic Leukemia. Leukemia.

[50] Shay J.W., Wright W.E. (2011). Role of Telomeres and Telomerase in Cancer. Semin. Cancer Biol..

[51] Counter C.M., Botelho F.M., Wang P., Harley C.B., Bacchetti S. (1994). Stabilization of Short Telomeres and Telomerase Activity Accompany Immortalization of Epstein-Barr Virus-Transformed Human B Lymphocytes. J. Virol..

[52] Counter C.M., Avilion A.A., LeFeuvre C.E., Stewart N.G., Greider C.W., Harley C.B., Bacchetti S. (1992). Telomere Shortening Associated with Chromosome Instability Is Arrested in Immortal Cells Which Express Telomerase Activity. EMBO J..

[53] Hemann M.T., Strong M.A., Hao L.-Y., Greider C.W. (2001). The Shortest Telomere, Not Average Telomere Length, Is Critical for Cell Viability and Chromosome Stability. Cell.

[54] Kyo S., Takakura M., Fujiwara T., Inoue M. (2008). Understanding and Exploiting *hTERT* Promoter Regulation for Diagnosis and Treatment of Human Cancers. Cancer Sci.

[55] McClintock B. (1942). The Fusion of Broken Ends of Chromosomes Following Nuclear Fusion. Proc Natl. Acad. Sci. USA.

[56] Barral A., Déjardin J. (2020). Telomeric Chromatin and TERRA. J. Mol. Biol..

[57] Kim W., Ludlow A.T., Min J., Robin J.D., Stadler G., Mender I., Lai T.-P., Zhang N., Wright W.E., Shay J.W. (2016). Regulation of the Human Telomerase Gene TERT by Telomere Position Effect-Over Long Distances (TPE-OLD): Implications for Aging and Cancer. PLoS Biol..

[58] Becker J.S., Nicetto D., Zaret K.S. (2016). H3K9me3-Dependent Heterochromatin: Barrier to Cell Fate Changes. Trends Genet..

[59] Nicetto D., Zaret K.S. (2019). Role of H3K9me3 Heterochromatin in Cell Identity Establishment and Maintenance. Curr. Opin. Genet. Dev..

[60] Jamieson K., Wiles E.T., McNaught K.J., Sidoli S., Leggett N., Shao Y., Garcia B.A., Selker E.U. (2016). Loss of HP1 Causes Depletion of H3K27me3 from Facultative Heterochromatin and Gain of H3K27me2 at Constitutive Heterochromatin. Genome Res..

[61] Nelson D.M., Jaber-Hijazi F., Cole J.J., Robertson N.A., Pawlikowski J.S., Norris K.T., Criscione S.W., Pchelintsev N.A., Piscitello D., Stong N. (2016). Mapping H4K20me3 onto the Chromatin Landscape of Senescent Cells Indicates a Function in Control of Cell Senescence and Tumor Suppression through Preservation of Genetic and Epigenetic Stability. Genome Biol..

[62] Benetti R., Gonzalo S., Jaco I., Schotta G., Klatt P., Jenuwein T., Blasco M.A. (2007). Suv4-20h Deficiency Results in Telomere Elongation and Derepression of Telomere Recombination. J. Cell Biol..

[63] Wiles E.T., Selker E.U. (2017). H3K27 Methylation: A Promiscuous Repressive Chromatin Mark. Curr. Opin. Genet. Dev..

[64] Guillemette B., Drogaris P., Lin H.-H.S., Armstrong H., Hiragami-Hamada K., Imhof A., Bonneil É., Thibault P., Verreault A., Festenstein R.J. (2011). H3 Lysine 4 Is Acetylated at Active Gene Promoters and Is Regulated by H3 Lysine 4 Methylation. PLoS Genet..

[65] Barski A., Cuddapah S., Cui K., Roh T.-Y., Schones D.E., Wang Z., Wei G., Chepelev I., Zhao K. (2007). High-Resolution Profiling of Histone Methylations in the Human Genome. Cell.

[66] Wendt K.S., Yoshida K., Itoh T., Bando M., Koch B., Schirghuber E., Tsutsumi S., Nagae G., Ishihara K., Mishiro T. (2008). Cohesin Mediates Transcriptional Insulation by CCCTC-Binding Factor. Nature.

[67] Dávalos-Salas M., Furlan-Magaril M., González-Buendía E., Valdes-Quezada C., Ayala-Ortega E., Recillas-Targa F. (2011). Gain of DNA Methylation Is Enhanced in the Absence of CTCF at the Human Retinoblastoma Gene Promoter. BMC Cancer.

[68] Luke B., Lingner J. (2009). TERRA: Telomeric Repeat-Containing RNA. EMBO J..

[69] Schoeftner S., Blasco M.A. (2008). Developmentally Regulated Transcription of Mammalian Telomeres by DNA-Dependent RNA Polymerase II. Nat. Cell Biol..

[70] Xu J., Kidder B.L. (2018). H4K20me3 Co-Localizes with Activating Histone Modifications at Transcriptionally Dynamic Regions in Embryonic Stem Cells. BMC Genom..

[71] Kychygina A., Dall’Osto M., Allen J.A.M., Cadoret J.-C., Piras V., Pickett H.A., Crabbe L. (2021). Progerin Impairs 3D Genome Organization and Induces Fragile Telomeres by Limiting the DNTP Pools. Sci Rep..

[72] Jost D., Vaillant C. (2018). Epigenomics in 3D: Importance of Long-Range Spreading and Specific Interactions in Epigenomic Maintenance. Nucleic Acids Res..

[73] Chuang T.C.Y., Moshir S., Garini Y., Chuang A.Y.-C., Young I.T., Vermolen B., van den Doel R., Mougey V., Perrin M., Braun M. (2004). The Three-Dimensional Organization of Telomeres in the Nucleus of Mammalian Cells. BMC Biol..

[74] Fabian-Morales E., Vallejo-Escamilla D., Gudiño A., Rodríguez A., González-Barrios R., Torres Y.L.R., Hernández C.C., Torre-Luján A.H., Oliva-Rico D.A., Guzmán E.C.O. (2021). Large-scale Topological Disruption of Chromosome Territories 9 and 22 Is Associated with Nonresponse to Treatment in CML. Int. J. Cancer.

[75] Zalensky A.O., Allen M.J., Kobayashi A., Zalenskaya I.A., Balhorn R., Bradbury E.M. (1995). Well-Defined Genome Architecture in the Human Sperm Nucleus. Chromosoma.

[76] Lange T. (1992). de Human Telomeres Are Attached to the Nuclear Matrix. EMBO J..

[77] Weierich C., Brero A., Stein S., von Hase J., Cremer C., Cremer T., Solovei I. (2003). Three-Dimensional Arrangements of Centromeres and Telomeres in Nuclei of Human and Murine Lymphocytes. Chromosome Res..

[78] Caria P., Dettori T., Frau D.V., Lichtenzstejn D., Pani F., Vanni R., Mai S. (2019). Characterizing the Three-dimensional Organization of Telomeres in Papillary Thyroid Carcinoma Cells. J. Cell. Physiol..

[79] Sunpaweravong S., Sunpaweravong P., Sathitruangsak C., Mai S. (2016). Three-dimensional Telomere Architecture of Esophageal Squamous Cell Carcinoma: Comparison of Tumor and Normal Epithelial Cells. Dis. Esophagus.

[80] Klewes L., Vallente R., Dupas E., Brand C., Grün D., Guffei A., Sathitruangsak C., Awe J.A., Kuzyk A., Lichtensztejn D. (2013). Three-Dimensional Nuclear Telomere Organization in Multiple Myeloma. Transl. Oncol..

[81] Knecht H., Sawan B., Lichtensztejn D., Lemieux B., Wellinger R.J., Mai S. (2009). The 3D Nuclear Organization of Telomeres Marks the Transition from Hodgkin to Reed–Sternberg Cells. Leukemia.

[82] Popova L.V., Nagarajan P., Lovejoy C.M., Sunkel B.D., Gardner M.L., Wang M., Freitas M.A., Stanton B.Z., Parthun M.R. (2021). Epigenetic Regulation of Nuclear Lamina-Associated Heterochromatin by HAT1 and the Acetylation of Newly Synthesized Histones. Nucleic Acids Res..

[83] The Human Genome Browser at UCSC. Genome.ucsc.edu.

[84] Kent W.J., Sugnet C.W., Furey T.S., Roskin K.M., Pringle T.H., Zahler A.M., Haussler D. (2002). The Human Genome Browser at UCSC. Genome Res..

[85] Galiveti C.R., Rozhdestvensky T.S., Brosius J., Lehrach H., Konthur Z. (2010). Application of Housekeeping NpcRNAs for Quantitative Expression Analysis of Human Transcriptome by Real-Time PCR. RNA.

[86] Whitaker N.J., Bryan T.M., Bonnefin P., Chang A.C., Musgrove E.A., Braithwaite A.W., Reddel R.R. (1995). Involvement of RB-1, P53, P16INK4 and Telomerase in Immortalisation of Human Cells. Oncogene.

[87] Hahn W.C., Counter C.M., Lundberg A.S., Beijersbergen R.L., Brooks M.W., Weinberg R.A. (1999). Creation of Human Tumour Cells with Defined Genetic Elements. Nature.

[88] Liu H.-T., Li F., Luan R.-H., Xing J.-L., Wang R.-A., Guo W.-Y., Wang H. (2009). Telomerase Activity Is Not Enough for Tumor Initiation in Human Cells. Afr. J. Biotechnol..

[89] Galigniana N.M., Charó N.L., Uranga R., Cabanillas A.M., Piwien-Pilipuk G. (2020). Oxidative Stress Induces Transcription of Telomeric Repeat-Containing RNA (TERRA) by Engaging PKA Signaling and Cytoskeleton Dynamics. Biochim. Biophys. Acta Bba. Mol. Cell. Res..

