# Supplementary material for: Methylation of Subtelomeric Chromatin Modifies the Expression of the lncRNA TERRA, Disturbing Telomere Homeostasis
Source: Int J Mol Sci. 2022 Mar 18;23(6):3271. doi: 10.3390/ijms23063271 (PMC8955364; doi:10.3390/ijms23063271)
Supplement: Supplementary file 1 [file ijms-23-03271-s001.zip › ijms-1618448-supplementary.pdf]

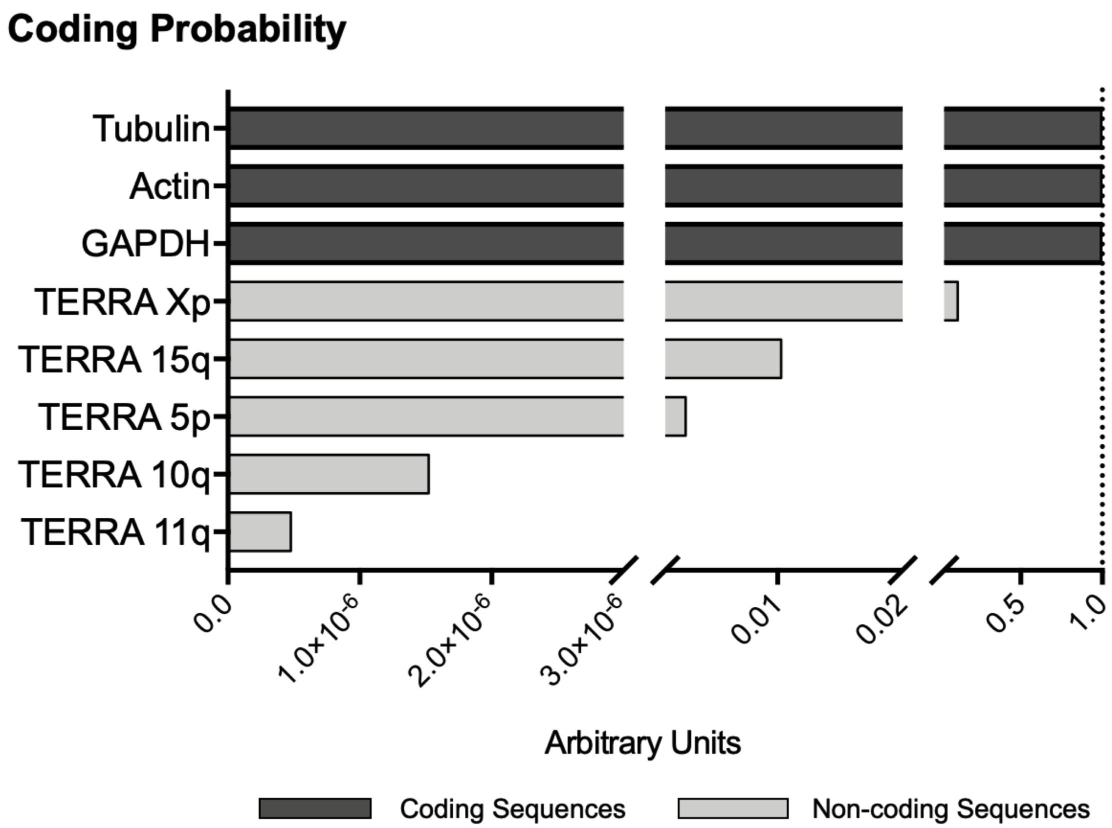

Figure S1. The analyzed TERRA loci do not have any coding potential. The coding probability of the analyzed sequences was verified using the coding potential calculator of the Center for Bioinformatics, Peking University, Beijing (http://cpc2.gao-lab.org/index.php). The *in silico* analysis confirmed that none of the sequences amplified using the listed TERRA primers (Supplementary Table 1) had neither ORF integrity nor any coding probability.

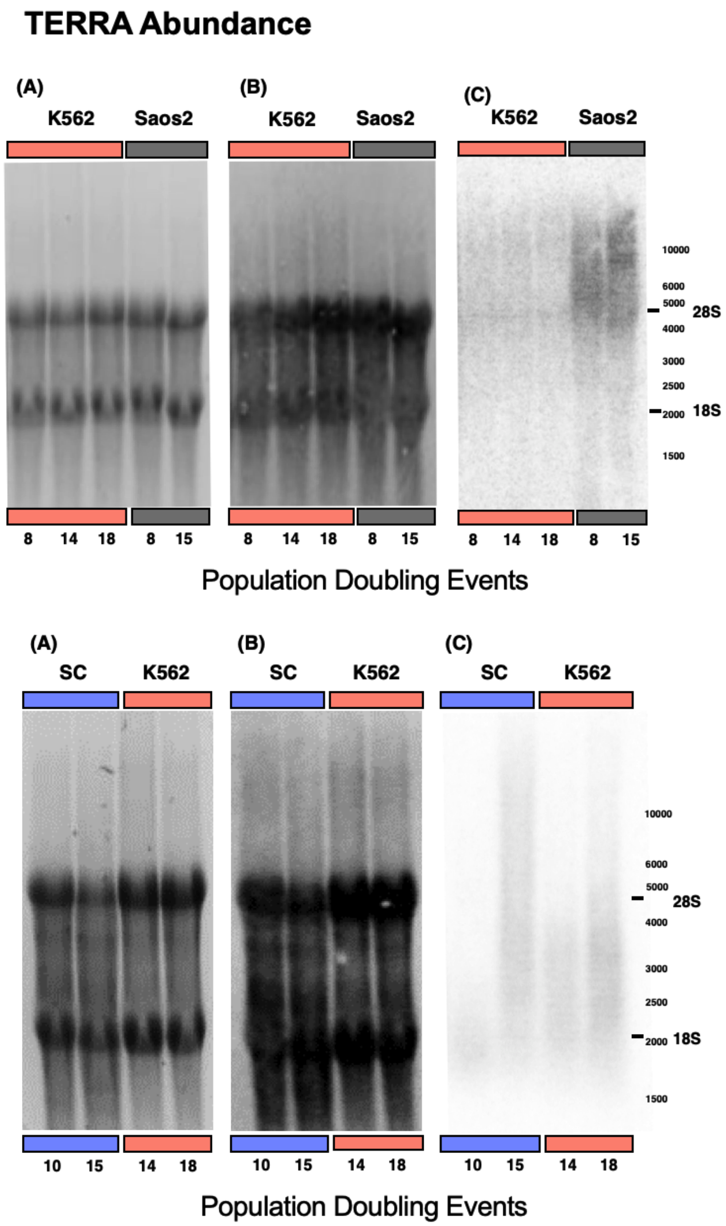

Figure S2. Northern blot for analysis of TERRA abundance. (Upper panel) TERRA abundance in different passages of K562 and Saos2 cells. (Lower panel) TERRA abundance in different passages of SC and K562 cells. In both panels: (A) Agarose gel after RNA electrophoresis. (B) Nylon membrane after RNA blotting. (C) Autoradiography after dCTP[P32]-labeled probe hybridization.

Quantitative-FISH Analysis

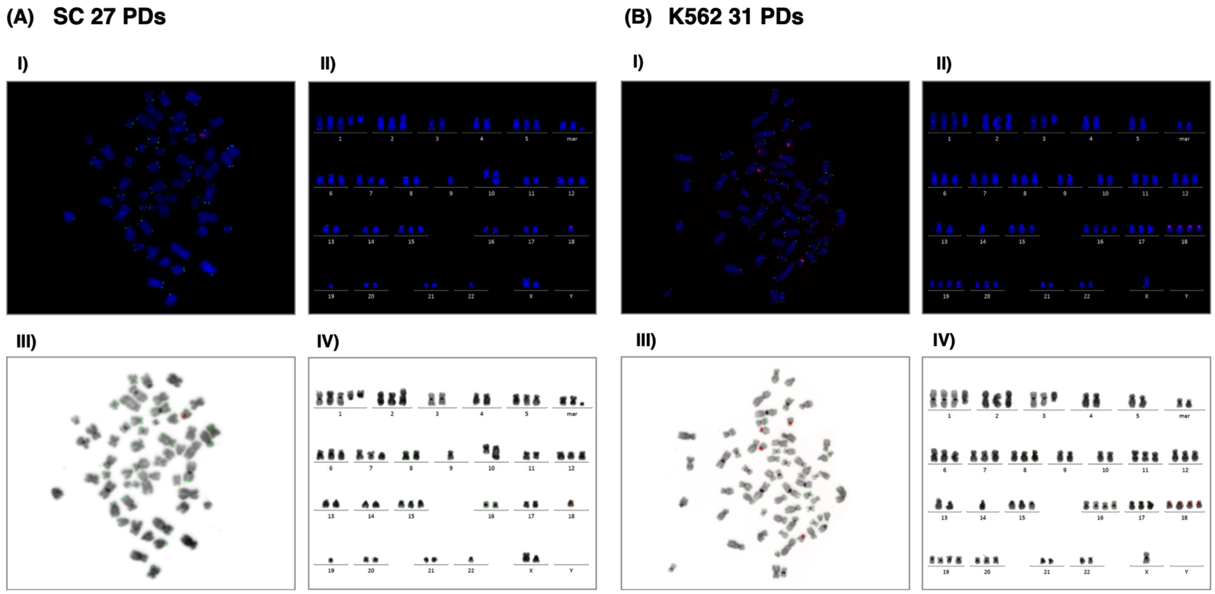

Figure S3. Metaphases and Karyograms for Q-FISH analysis. (A) SC cells after 27 population doubling events. (Upper panel) I) Metaphase hybridized with a pant-telomeric FitC probe, a centromeric Cy5 probe for chromosome 18, and counterstained with DAPI. II) Karyogram of I). (Lower panel) III) Grayscale-converted DAPI counterstain of the metaphase shown in I). IV) Negative/False color of the karyogram shown in II). (B) K562 cells after 31 population doubling events. (Upper panel) I) Metaphase hybridized with a pant-telomeric FitC probe, a centromeric Cy5 probe for chromosome 18, and counterstained with DAPI. II) Karyogram of I). (Lower panel) III) Grayscale-converted DAPI counterstain of the metaphase shown in I). IV) Negative/False color of the karyogram shown in II).

Probes for Northern Blot				
**Target**	**Probe**	**Sequence (5' -3')**		
U6	Rv	CGCTTCACGAATTTGCGTGTCATCCTTGCGCAGGGGCCATGC		
TERRA	Rv	CCCTAACCCTAACCCTAACCCTAACCCTAACCCTAACCCTAACC		
**Primers for qPCR analysis**				
**Transcript**	**Primer**	**Sequence (5' -3')**	**Known Loci**	**Reference**
5p-TERRA	Fw	CGGTGTACAGGATTCACAGGG	1 (5p)	-
	Rv	GCTTTGGGACAACTCGAGGC		
10q-TERRA	Fw	GCCTTGCCTTGGGAGAATCT	7 (1q, 2q, 4q, 10q, 13q, 21q, 22q)	[59]
	Rv	AAAGCGGGAAACGAAAAGC		
11q-TERRA	Fw	CAGACCTTGGAGGCACGGCCTTCG	1 (11q)	-
	Rv	CCCTGATTATTCAGGGCTGCAAAG		
15q-TERRA	Fw	TGCAACCGGGAAAGATTTTATT	5 (1q, 8p, 15q, 16p, 21q, 22q)	[59]
	Rv	GCGTGGCTTTGGGACAACT		
Xp-TERRA	Fw	GAGTGAAAGAACGAAGCTTCC	4 (Xp (2), Yp (2))	-
	Rv	CCCTCTGAAAGTGGACCTAT		
U6	Fw	CTCGCTTCGGCAGCACA	na	[60]
	Rv	AACGCTTCACGAATTTGCG		
hTERT	Fw	AGGGGCAAGTCCTACGTCCAGT	na	[10]
	Rv	CACCAACAAGAAATCATCCACC		
GAPDH	Fw	ACCCACTCCTCCACCTTTG	na	-
	Rv	CTCTTGTGCTCTTGCTGGG		
**Primers for ChIP analysis**				
**Transcript**	**Primer**	**Sequence (5' -3')**	**Histone Mark Enrichment**	
**p53 Promoter**	Fw	TGCTGGGAGTTGTAGTCTG	CTCF	
	Rv	GATGCAGAAGAGGTGCAAG		
**GAPDH Exons 5-6**	Fw	GGAGTCCACTGGCGTCTTC	H3K4me3	
	Rv	GGTTCACACCCATGACGAAC		
**GAPDH 5'UTR-Exon 1**	Fw	GTTTCTATAAATTGAGCCCGCAG	Ser5-P RNA Pol 2	
	Rv	AAAAGAAGATGCGGCTGACTG		
**MYT1 5'UTR**	Fw	CACCCCCAATTCAGCAGAT	H3K9me3	
	Rv	TACGCTACCCATGAACCCT		
**OCT4/POU5F1 Exon 1**	Fw	AGCTGCCCACCTAACTTCTA	H4K20me3	
	Rv	AGGGTCTCTCTTTCTGTCCT		
**SOX2-OT Intron**	Fw	CTTTCAGTTTCGTTGCTCCAACCCTGAC	H3K27me3	
	Rv	CCCTTCTTCCCTTTTTGCTGGCTCTCC		

Table S1. List of the used primers and probes and their sequences. The primers for TERRA expression analysis include other loci that have been previously reported.
